# The role of eosinophils and their activation state in hypereosinophilia-associated heart disease

**DOI:** 10.3389/fimmu.2025.1635483

**Published:** 2025-09-19

**Authors:** Usman Sunusi, Immaculeta Osuji, Benjamin Ziegelmeyer, Mario Medvedovic, Haley Todd, Joe Abou-Khalil, Nives Zimmermann

**Affiliations:** ^1^ Department of Pathology and Laboratory Medicine, University of Cincinnati College of Medicine, Cincinnati, OH, United States; ^2^ Department of Pharmacology, Physiology, and Neurobiology, University of Cincinnati College of Medicine, Cincinnati, OH, United States; ^3^ Department of Biostatistics, Health Informatics and Data Sciences, University of Cincinnati College of Medicine, Cincinnati, OH, United States; ^4^ Department of Molecular, Cellular, and Developmental Biology, Yale University, New Haven, CT, United States

**Keywords:** eosinophil-associated disease, myocarditis, single cell RNA sequencing, PDL1, eosinophil

## Abstract

**Background:**

Cardiac complications in patients with hypereosinophilia cause significant morbidity and mortality. However, mechanisms of how eosinophilic inflammation causes heart damage are poorly understood.

**Methods:**

We developed a model of hypereosinophilia-associated heart disease by challenging hypereosinophilic mice with a peptide from the cardiac myosin heavy chain. Disease outcomes were measured by histology, immunohistochemistry, flow cytometry, and measurement of cells and biomarkers in peripheral blood. Eosinophil dependence was determined by using eosinophil-deficient mice (ΔdblGATA). Single cells from the heart were subjected to single-cell RNA sequencing to assess cell composition, activation states, and expression profiles. *In vitro* studies used bone marrow-derived eosinophils (BMDeos) and stimulated them with cytokines and pathogen-associated molecular patterns, followed by assessment of activation markers by flow cytometry.

**Results:**

Mice challenged with the myocarditic and control peptide had peripheral blood leukocytosis, but only those challenged with the myocarditic peptide had heart inflammation. Heart tissue was infiltrated by eosinophil-rich inflammatory infiltrates associated with cardiomyocyte damage. Disease penetrance and severity were decreased in eosinophil-deficient mice. Single-cell RNA sequencing showed the enrichment of myeloid cells, T cells, and granulocytes (neutrophils and eosinophils) in myocarditic mice. Focusing on eosinophils, there was increased expression of genes associated with type 1 cell activation (such as CD274/PDL1), complement activation, and pathogen-associated molecular pattern recognition. To verify findings generated by single-cell RNA sequencing on a protein level, we performed flow cytometry analysis and assessed the level of type 1 and type 2 biomarkers CD274 and CD101, respectively. The proportion of cells expressing surface CD274 increased on both neutrophils and eosinophils, particularly in mice that showed inflammation by histology. There was no significant increase in expression of CD101. Finally, we assessed whether activation markers can be induced on eosinophils *in vitro*. Interferon γ (IFNγ) markedly increased expression of CD274, consistent with type 1 polarization. Furthermore, BMDeos stimulated with LPS showed a concentration-dependent increase in the level of CD274 expression.

**Conclusion:**

Eosinophils are required for heart damage in hypereosinophilia-associated heart disease. Heart-infiltrating eosinophils in an inflammatory condition show type 1 activation, which can be recapitulated *in vitro*.

## Introduction

Eosinophil-associated disorders (EADs), including hypereosinophilic syndrome (HES), eosinophilic granulomatosis with polyangiitis (EGPA), and eosinophilic gastrointestinal disorders (EGIDs), are a heterogeneous group of conditions characterized by blood and/or tissue hypereosinophilia and eosinophil-related clinical manifestations (1). Cardiac complications occur in up to 60% of patients with sustained hypereosinophilia (2–4) and are a major cause of morbidity and mortality in this patient population. In patients with eosinophilic heart disease (EHD), the clinical course is characterized by eosinophil-rich endomyocarditis with cardiomyocyte necrosis, followed by replacement fibrosis in the myocardium and possibly thrombosis stemming from endocardial damage, eventually leading to cardiomyopathy (3–7).

As the NIH Taskforce on Research needs of Eosinophil-Associated Diseases (TREAD) and recent RE-TREAD reported, there is a paucity of preclinical models that adequately replicate cardiac disease in hypereosinophilia, and development of these models would enable mechanistic studies aiming to develop targeted therapies (8, 9). To address this unmet need, we have recently developed (10, 11) a mouse model of EHD that recapitulates many of the salient features of human disease importantly, including hypereosinophilia with heart involvement reminiscent of that seen in patients. While informative, this model has several limitations including low and highly variable penetrance, unpredictable clinical course, and first presentation with sudden death. These limitations make mechanistic studies difficult. Diny et al. (12) challenged wild-type and hypereosinophilic mice with cardiac myosin peptide to induce experimental autoimmune myocarditis (EAM) and eosinophilic EAM (eoEAM). They showed that the progression of myocarditis to dilated cardiomyopathy (DCM) is dependent on the presence of eosinophils, thus implicating them in the pathophysiology of disease. While this progression was dependent on eosinophil production of intereleukin-4 (IL-4) in the EAM model (not associated with hypereosinophilia), the mechanism of eosinophil-mediated disease effects has not been studied in hypereosinophilic mice (eoEAM model), which showed significantly different levels of inflammation and cardiac dysfunction (12). Notably, studies in eosinophil-associated diseases (beyond heart disease) have shown that eosinophils may contribute to either tissue repair or tissue damage, which is likely disease-dependent. Therefore, the focus of studies presented in this manuscript was to study the mechanism of eosinophil-mediated effects on heart function in hypereosinophilia, specifically which role eosinophils play.

Furthermore, recent studies have shown eosinophil heterogeneity between and within organs in homeostasis (13, 14) and disease processes. Because majority of the work on eosinophil phenotypes has focused on lungs and the gastrointestinal tract, the cardiac eosinophil phenotype is not well understood. Therefore, in this manuscript, we focused on the activation state of eosinophils in cardiac inflammation.

## Methods

### Mice

IL-5 transgenic (IL-5tg) mice in which the IL-5 gene is driven by the CD2 promoter (15) on a BALB/c background were provided by Dr. Marc Rothenberg (Cincinnati Children’s Hospital). Eosinophil-deficient ΔdblGATA mice (16) (BALB/c background) were provided by Dr. Rothenberg, with approval from Dr. Orkin. Mice were housed in a specific pathogen-free facility at the University of Cincinnati. Experiments were conducted on >6-week-old mice of both genders; initial studies did not identify gender-specific differences in measured outcomes. All procedures and protocols were approved by the Institutional Animal Care and Use Committee of the University of Cincinnati.

To induce myocarditis, IL-5tg received subcutaneous immunizations on days 0 and 7 of 100 μg of myosin heavy chain α (MyHCα) 614 (myocarditic) peptide (Ac-SLKL MATL FSTY ASAD; Genscript) or 790 (non-myocarditic control) peptide (Ac-IQAQ ARGQ LMRI EFKK) (17) emulsified in complete Freund adjuvant (CFA, Sigma-Aldrich) supplemented with 5 mg/mL heat-killed *Mycobacterium tuberculosis* strain H37Ra (BD Biosciences). On day 0, mice also received 500 ng of pertussis toxin intraperitoneally (List Biologicals) (18).

Pre-challenge and weekly during the protocol, venous blood was collected from mice via submandibular puncture. For complete blood counts, blood was collected in K_2_EDTA-coated tubes (BD Biosciences), while for serum, blood was collected in tubes coated with a clot accelerator and serum separator gel (BD Biosciences). Samples were inverted in tubes to mix with coating and allowed to settle for 30–60 min. The sample was then centrifuged at 1,000*g* for 10 min at 4°C. Sera were aliquoted to sterile tubes and stored at –80°C until use in assay.

At sacrifice, the hearts were perfused with DPBS (Gibco) supplemented with 0.9 mm of CaCl_2_ (Alfa Aesar) and collected for single-cell suspension preparation, histology, and/or RNA isolation.

### Cell-free DNA in serum

Sera were warmed to room temperature and diluted 1:20 in assay buffer 30–60 min before assay. Quantification of cell-free dsDNA was performed by fluorometry using PicoGreen assay following the manufacturer’s instructions (Quant-iT PicoGreen dsDNA Kit; Invitrogen). Plates were read on a GloMax Multi Detection System (Promega) at wavelengths of 480 and 520 nm for excitation and emission, respectively. Fluorescence values were subtracted from sample/standard curve fluorescence values, and concentration of cell-free DNA (cfDNA) was calculated from the standard curve. All steps of the assay were performed at room temperature.

### Troponin

Sera were thawed to room temperature and diluted 1:10 in an assay diluent 30 min before assay. Quantification of cardiac troponin-I was performed by spectrophotometry using a Mouse Cardiac Troponin-I ELISA Kit following the manufacturer’s instructions (CTNI-1-US; Life Diagnostics). Absorbance of wells was measured on a GloMax Multi Detection System (Promega) at a wavelength of 450 nm. Blank absorbance values were subtracted from sample/standard curve absorbance values, and the concentration of cardiac troponin-I was calculated from the standard curve. All steps of the assay were performed at room temperature.

### Flow cytometry

Single-cell suspensions were made from hearts, and bone marrow-derived eosinophils (BMDeos) were cultured and stimulated as below. For immunophenotyping, cells were stained with a panel of antibodies that included SiglecF-PE (BioLegend), CD19-APC (Invitrogen), CD3-FITC (Invitrogen), Ly6G-BV421 (BD Horizon), CD45-APCy7 (BD Pharmingen), and 7AAD (Bioscience). When staining for markers of activation, we used CD274-APC (BioLegend) and CD101-AlexaFluor700 (eBioscience). Data were collected on a Canto3 or LSR Fortessa flow cytometer. Compensation, settings, and gating are described in the **Supplementary Materials** (MIFlowCyt format).

### Histology

Tissues were fixed in formalin and embedded into paraffin blocks. Sections were stained with hematoxylin and eosin (H&E) and Trichrome using standard techniques at the Pathology core at Cincinnati Children’s hospital. Anti-MBP immunohistochemistry was performed with antibody gifted by Dr. Elizabeth Jacobsen (Mayo Clinic) using established methods (19).

### Peripheral blood cell counts

Peripheral blood was collected in EDTA-coated tubes and complete blood counts [absolute white blood cell, red blood cells (RBCs), and platelet count] performed using an automated cell counter (Heska). A peripheral blood smear was prepared and stained using Diff Quick (Epredia), and manual differential cell count was performed (since eosinophil count was inaccurate on an automated cell counter). The absolute count of individual cell types was calculated from the absolute white blood cell count from an automated counter and manual differential count.

### Single-cell suspensions

The heart single-cell suspensions were prepared as per 10x Genomics single-cell protocol (CG00053 Rev C). Briefly, the hearts were cut into halves using a four-chamber cut, and half a heart was saved for histology, while the other half was used for the preparation of single-cell suspension. Heart tissue was minced, followed by enzymatic digestion (2.2 mg/mL Collagenase IV, Worthington and 1.5 mg/mL Dispase II, Life Technologies) at 37°C for 45 min. Subsequently, samples were filtered through a 40-µm filter, and RBCs were lysed (eBioscience RBC Lysis Buffer, Thermo Fisher Scientific). Cells were then resuspended in RPMI supplemented with 10% fetal bovine serum (FBS) and filtered through a 30-µm MACS cell strainer (MACS Filters, Miltenyi Biotec). The cells were counted to determine the concentration and viability using a hemocytometer, after which they were subsequently fixed for chromium fixed RNA profiling.

### Fixation of heart single-cell suspension for chromium fixed RNA profiling

The fixation was done following the 10x Genomics Fixation of Cells & Nuclei for Chromium Fixed RNA Profiling protocol (CG000478 | Rev C). Briefly, the heart single-cell suspensions were centrifuged at 400*g* and resuspended in Fixation Buffer, followed by storage at 4°C for 16–24 h. Following centrifugation at 850*g* for 5 min at room temperature (22°C), the sample pellet is resuspended in chilled Quenching Buffer, and cell concentration is determined. Pre-warmed Enhancer and glycerol (Thermo Fisher Scientific) are added prior to storing cells at −80°C.

### Workflow for single-cell RNA-seq data processing and analysis

For each mouse, half of the heart was fixed for histology and the other half was used for single-cell suspension and fixation in a Chromium flex kit and stored at –80°C. Once histological assessment was performed (by an observer blinded to treatment), we selected which mice to subject to single-cell RNA sequencing (scRNA-seq). Two mice challenged with the myocarditic peptide who had pancarditis by histology and one mouse challenged with the control peptide who had no inflammation were selected. Additional criteria included number and viability of cells in the single-cell suspension preparation. Library preparation and sequencing were performed at the Genomics Sequencing Facility at Cincinnati Children’s Hospital (Core Marketplace Research Resource Identifier RRID: SCR_022630). The fixed RNA profiling assay was performed according to the manufacturer’s instructions (Chromium Fixed RNA Profiling Reagent Kit User Guide, 10x Genomics). Briefly, individual suspensions of fixed cells were subjected to sample barcoding (BC001–BC004) using the Chromium Fixed RNA Kit, Mouse Transcriptome (PN-1000496). Mouse whole transcriptome probe pairs were used for overnight probe hybridization. Next, the barcoded samples were pooled, washed, and subjected to gel bead-in-emulsion (GEM) generation using the Chromium Next GEM Single Cell Fixed RNA Sample Preparation Kit (PN-1000414) and the Chromium Next GEM Chip Q Single Cell Kit (PN-1000418/PN-1000422). The cells were resuspended in a master mix and loaded together with partitioning oil and gel beads into the chip to generate GEMs. Upon entering a droplet, the gel beads dissolved, releasing single-cell barcoding primers, and the fixed cells were lysed, exposing the RNA with probe pairs hybridized to it. The GEMs were collected and incubated in a thermocycler, allowing ligation of the probe pairs followed by hybridization and incorporation of the single-cell barcoding primers. The single-cell barcoding primers incorporated partial Read 1T, a 16-nucleotide 10x GEM Barcode, a 12-nucleotide unique molecular identifier (UMI), and partial Capture Sequence 1 to the ligated probe pairs. Next, the GEMs were broken, and the cell-barcoded molecules were cleaned up with Silane DynaBeads, then subjected to pre-amplification and SPRIselect reagent size selection. Finally, a gene expression library was constructed. P5, P7, i5, and i7 sample indexes; Illumina TruSeq Read 1 sequence (Read 1T); and Small Read 2 (Read 2S) sequences were added to generate Illumina sequencer-ready libraries using the Dual Index Kit TS Set A (PN-1000251). The samples were run on one lane of a 10B flow cell on the NovaSeq X Plus sequencer with the following sequencing parameters: R1: 28 cycles, i7: 10 cycles, i5: 10 cycles, and R2: 90 cycles.

The Cell Ranger software package from 10x Genomics v.8.0 was utilized to process the raw FASTQ files generated from scRNA-seq and aligned the sequencing reads to a mouse mm10 reference genome. Additionally, the Cell Ranger performed the initial filtering of low-quality or empty droplets to retain valid cells, as well as filtering genes based on expression levels to focus on those with significant expression that ensured high-quality filtered data ready for downstream analysis and interpretation in Seurat. Raw and processed data have been deposited in the GEO repository under accession number GSE295865.

The Seurat package v5.1.0 was used to preprocess and analyze single-cell RNA-seq data obtained from Cell Ranger. First, the dataset was filtered to retain cells with more than 100 RNA counts and less than 15% mitochondrial gene expression to eliminate potential low-quality cells. The data were normalized, and highly variable features were identified. Subsequently, the data were scaled, and principal component analysis (PCA) was performed. Dimensional reduction to form the uniform manifold approximation and project (UMAP) utilized the top 20 calculated dimensions. The Doublet Finder package V. 2.0.4 was used to remove doublets (20) using 8% expected doublet rate formation parameter. Samples were normalized using the sctransform approach with default settings (21). Dimensional reduction was then performed using the UMAP, and the top 30 calculated dimensions and a resolution of 0.2 were utilized. Data integration was performed in Seurat (22, 23) to merge the single-cell RNA-seq datasets from different conditions facilitating joint analysis. It identifies common features across datasets to align and correct technical differences, enabling the comparison and analysis of cells from disparate sources. Clusters were annotated using a combination of canonical markers of cell lineages provided in **Supplementary Table S1**, the SingleR (v2.8.9) R package (24) with correlations of the single-cell expression values with transcriptional profiles from pure cell populations in the Immgen database (25), and the FindAllMarker function in Seurat.

Differential gene expressions were assessed using the Wilcoxon rank-sum test on count-level mRNA data. For differential comparisons within a cluster across conditions, the FindAllMarkers function from the Seurat package was applied to the integrated SCTransform-normalized dataset, using a log-fold change threshold of >0.2, a minimum detection percentage of 10%, and a minimum percentage difference of 0% between groups. The differentially expressed gene (DEG) lists were further refined using adjusted *p*-value <0.05 and fold change >/<1 for upregulated/downregulated genes (DEG lists for eosinophils, neutrophils, and mono/mac/DC are provided in the **Supplementary Table 2**), in order to perform Gene Ontology (GO) enrichment analysis to identify overrepresented biological processes and analysis of eosinophil subsets/activation states (14). The analysis was conducted using R with the clusterProfiler (v.4.3.1) (26), org.Mm.eg.db (v.3.17.0), and Annotation (v.1.64.1).

### RNA isolation and cDNA synthesis

Heart halves (5–8 mg) that were harvested from myocarditis and control mice were placed in Trizol (Ambion; Life Technologies), homogenized, and stored at −80°C. For the isolation of RNA from single-cell suspensions, 0.5–1 × 10^6^ cells were collected by centrifugation and lysed with RA1 buffer from the NucleoSpin RNA kit (Macherey-Nagel) and β-mercaptoethanol (Acros Organics). RNA was then isolated using the NucleoSpin RNA kit following the manufacturer’s instructions. The purified RNA was quantified using the NanoDropND-1000 spectrophotometer (NanoVue Plus) stored at −80°C until further use. Total RNA was reverse-transcribed into cDNA using the Invitrogen SuperScript IV kit (Thermo Fisher Scientific) using Oligo dT primers and including reverse transcriptase-negative (RT−) control. The resulting cDNA samples were used for quantitative polymerase chain reaction (qPCR) either immediately or following storage at −20°C.

### RNA amplification via qPCR

qPCR was performed using the Maxima SYBR Green qPCR Master Mix (Thermo Fisher Scientific). Specific primer sequences were as follows: Arg1 Forward: ACACTCCCCTGACAACCAGC; Arg1 Reverse: AGGGTCTACGTCTCGCAAGC; Prg2 Forward: TTGCAAACTTGACAAGACCCAGG; Prg2 Reverse: CCCCCGACTAGAAGAGCCAGA; Il1a Forward: TGAAGCTCGTCAGGCAGAAGT; Il1a Reverse: TCCTCCCGACGAGTAGGCAT; Pecam1 Forward: GAGCCTCACCAAGAGAACGG; Pecam1 Reverse: AGCGCCTCTGAGTCTCTGTA; Actb Forward: AGCTCCTTCGTTGCCGGT; Actb Reverse: ACCCATTCCCACCATCACACC. The qPCR reaction was performed using the qPCR machine (Bio-Rad CFX) with the following parameters: initial denaturation step was performed at 95°C for 10 min, followed by 39 cycles of 95°C for 15 s, and annealing at 58°C for 1 min. Following quality control review (amplification and melting curves), relative gene expression is presented using δδCt analysis.

### Bone marrow-derived eosinophil generation and stimulation

Derivation of BMDeos was performed as previously described (27). In brief, bone marrow cells were collected by flushing the femurs and tibiae, with medium (RPMI with 20% FBS and HEPES), using a 21-gauge needle, and filtering through a sterile 70-μm nylon cell strainer. After RBC lysis, cells were reconstituted at 1 × 10^6^/mL and incubated at 37°C in 100 ng/mL of SCF and FLT3L for 4 days, followed by 10 ng/mL of IL-5 for an additional 10 days. Cells were stimulated at 1 × 10^6^ cell/mL for 18 h with LPS (10, 50, and 100 ng/mL) and interferon γ (IFNγ) (50 ng/mL), washed, and subjected to staining for flow cytometry as described above and in the **Supplementary Materials**.

## Results

### Eosinophilic experimental autoimmune myocarditis

**Figure 1:**
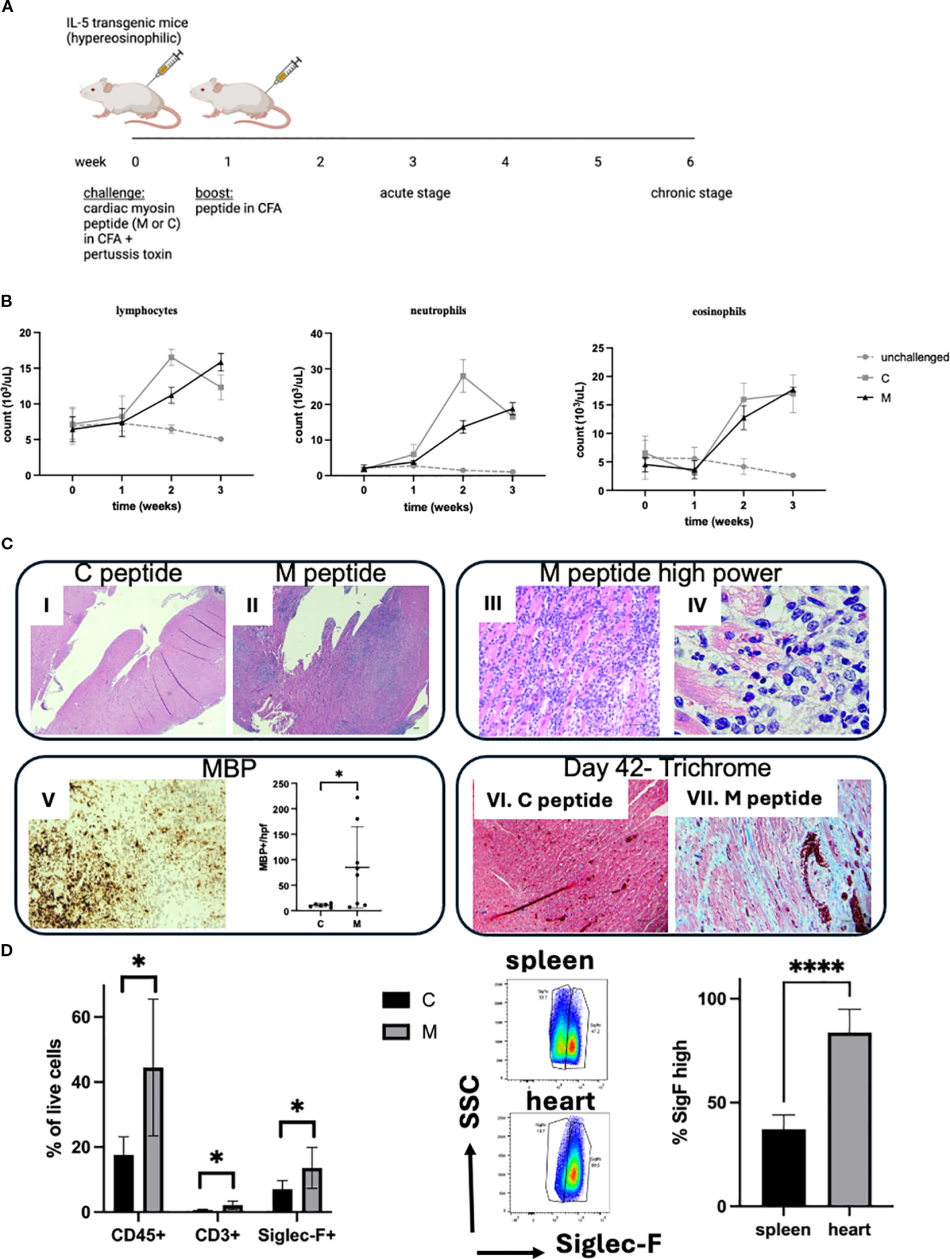
Hypereosinophilia-associated heart disease model. **(A)** Schematic representation of the model is shown. M, myocarditic peptide; C, control peptide; CFA, complete Freund adjuvant. **(B)** White blood cells were counted using an automated cell counter followed by manual differential on peripheral blood smears from unchallenged (gray dotted line), and mice challenged with control (C, gray solid line) and myocarditic (M, black solid line) peptide. Shown are lymphocytes, neutrophils, and eosinophils. Data are average ± SD from three experiments with 2, 13, and 15 mice total (unchallenged, C and M, respectively). **(C)** Histologic assessment from the heart is shown: (I, II): H&E-stained heart from mice challenged with C and M peptide, respectively, 21 days after challenge, at 40× magnification (scale bar, 100 μm); (III, IV): H&E-stained heart from mouse challenged with M peptide at higher magnification ((III): 400×, scale bar, 50 μm; IV: 1000× oil, scale bar, 20 μm); (V): anti-MBP stained heart from representative mouse challenged with M peptide (200×, scale bar, 50 μm) and quantification of MBP-positive cells per peak field at 1000× oil; (VI, VII): Trichrome-stained hearts from mice challenged with C (VI) and M (VII) peptide on day 42 (200×, scale bar, 50 μm). Representative mice from 48 and 100 mice challenged with control and myocarditic peptide respectively are shown. **(D)** Heart cells were analyzed by flow cytometry. Single-cell suspensions of hearts from mice challenged with control **(C)** and myocarditic (M) peptide were subjected to flow cytometry for hematopoietic cells (CD45+), T cells, (CD3+), eosinophils (SiglecF+), B cells (CD19+, data not shown), and neutrophils (Ly6Ghi/SiglecF-, data not shown). In the middle panel, the level of Siglec-F is compared in spleen and heart eosinophils in mice challenged with M peptide, and % Siglec-F high cells plotted. *p < 0.05; ****p < 0.0001. Data are from six to seven mice per group.

In order to mimic hypereosinophilia-associated heart disease, we adapted the EAM model. Hypereosinophilic mice (CD2.IL5tg) were challenged with a myocarditic (M) and non-myocarditic control (C) peptide from cardiac alpha myosin (**Figure 1A**). Following antigen challenge, mice developed peripheral blood leukocytosis, represented by lymphocytosis, neutrophilia, and eosinophilia (comparable between two peptides since both peptides were emulsified in CFA, **Figure 1B**). However, on day 21, while mice challenged with the control peptide do not show heart inflammation, heart histology in the majority of mice challenged with the myocarditic peptide showed inflammation of all three layers—endocardium, myocardium, and epicardium (**Figure 1C**)—which was eosinophil-rich (highlighted by anti-MBP staining) and associated morphologically with cardiomyocyte death (**Figure 1C**). From 19 experiments performed to date with a total of 100 mice challenged with the myocarditic peptide, the average disease penetrance (percent of mice with heart inflammation by histology) is 59%. In contrast, none of the mice challenged with the control peptide (*n* = 48) developed myocarditis (*p* < 0.0001, Fisher’s exact test). No difference between male and female mice was seen in penetrance or severity of disease. Furthermore, analysis of other organs failed to reveal any destructive inflammation in kidneys, spleen, liver, or skeletal muscle (data not shown). In a separate experiment, mice were sacrificed on day 42, and those challenged with the myocarditic peptide histologically showed increased fibrosis (**Figure 1C**). Flow cytometry of cardiac single-cell suspensions showed increased proportion of hematopoietic cells (CD45+), T cells (CD3+), and eosinophils (Siglec-F+) (**Figure 1D**), with no statistically significant difference in neutrophils and B cells (data not shown). Furthermore, based on the level of surface Siglec-F, eosinophils in spleen show two populations with large Siglec-F low and smaller Siglec-F high (activated population), while eosinophils in the heart were uniformly activated, Siglec-F high (**Figure 1D**).

**Figure 2:**
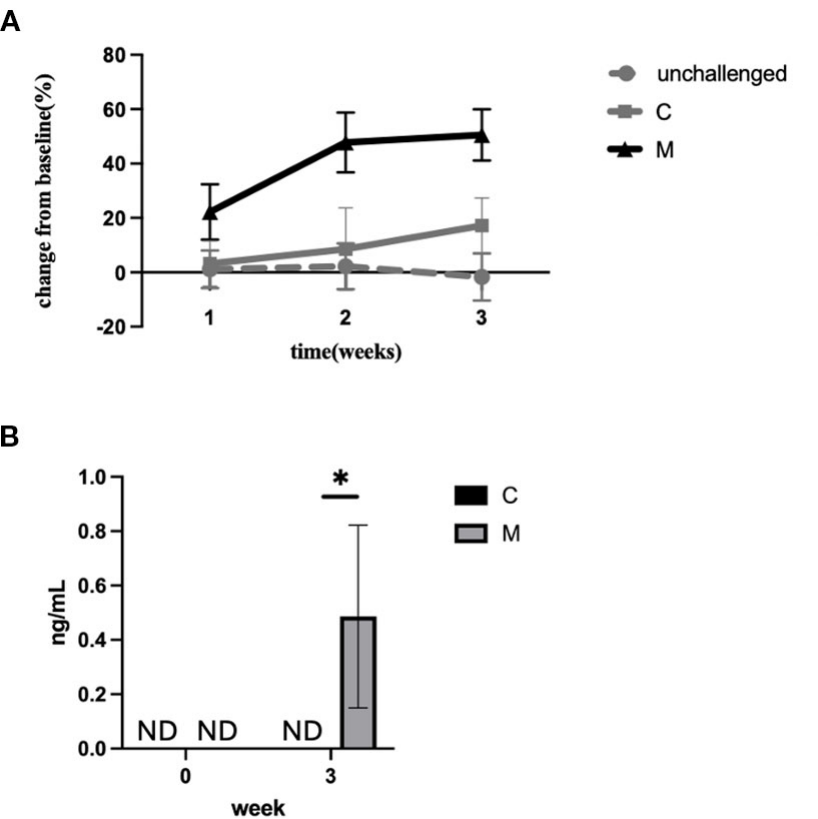
Tissue damage in the eoEAM model. **(A)** cfDNA was measured in the serum of mice challenged with the M peptide (black solid line), C peptide (gray solid line), or unchallenged mice (gray dotted line) over 3 weeks of challenge. Data are shown for % change over baseline for each mouse, with mean and SD of three experiments (with three to four mice per group in each experiment). *p* < 0.0001 by two-way ANOVA. **(B)** Troponin was measured in the serum of mice challenged with myocarditic (M) or control (C) peptide at baseline and peak inflammation time point (week 3). *p < 0.05. ND = not detectable.

Histologic assessment showed cardiomyocyte dropout and damaged cardiomyocytes, some with clear eosinophil-free extracellular granules (e.g., **Figures 1C-iv**). Thus, we hypothesized that there is tissue (particularly cardiomyocyte) damage. In order to assess for signs of tissue damage, we measured the level of cfDNA circulating in peripheral blood (**Figure 2A**). The level of circulating cfDNA did not change significantly over time in unchallenged mice or mice challenged with the C peptide. However, the cfDNA levels increased 22.2 ± 10.2-fold at 1 week, 47.8 ± 11-fold at 2 weeks, and 50.5 ± 9.5-fold at 3 weeks (*p* < 0.0001 by two-way ANOVA) in mice challenged with the M peptide. In order to assess for cardiomyocyte damage, we measured the level of cardiac troponin in the serum of mice. The level of troponin was undetectable at baseline, and increased to measurable level at 3 weeks in mice challenged with the M peptide (**Figure 2B**). In contrast, unchallenged mice and C-peptide-challenged mice did not have measurable troponin. Together, these data indicate that mice challenged with the myocardiatogenic peptide experience cardiomyocyte damage.

In summary, the EHD model shows eosinophil-rich inflammation associated with cardiomyocyte necrosis (early) and dropout with replacement fibrosis (late). Thus, we now have a model to most efficiently and thoroughly test the role of eosinophils in heart inflammation associated with hypereosinophilia.

### The role for eosinophils in heart inflammation

**Figure 3:**
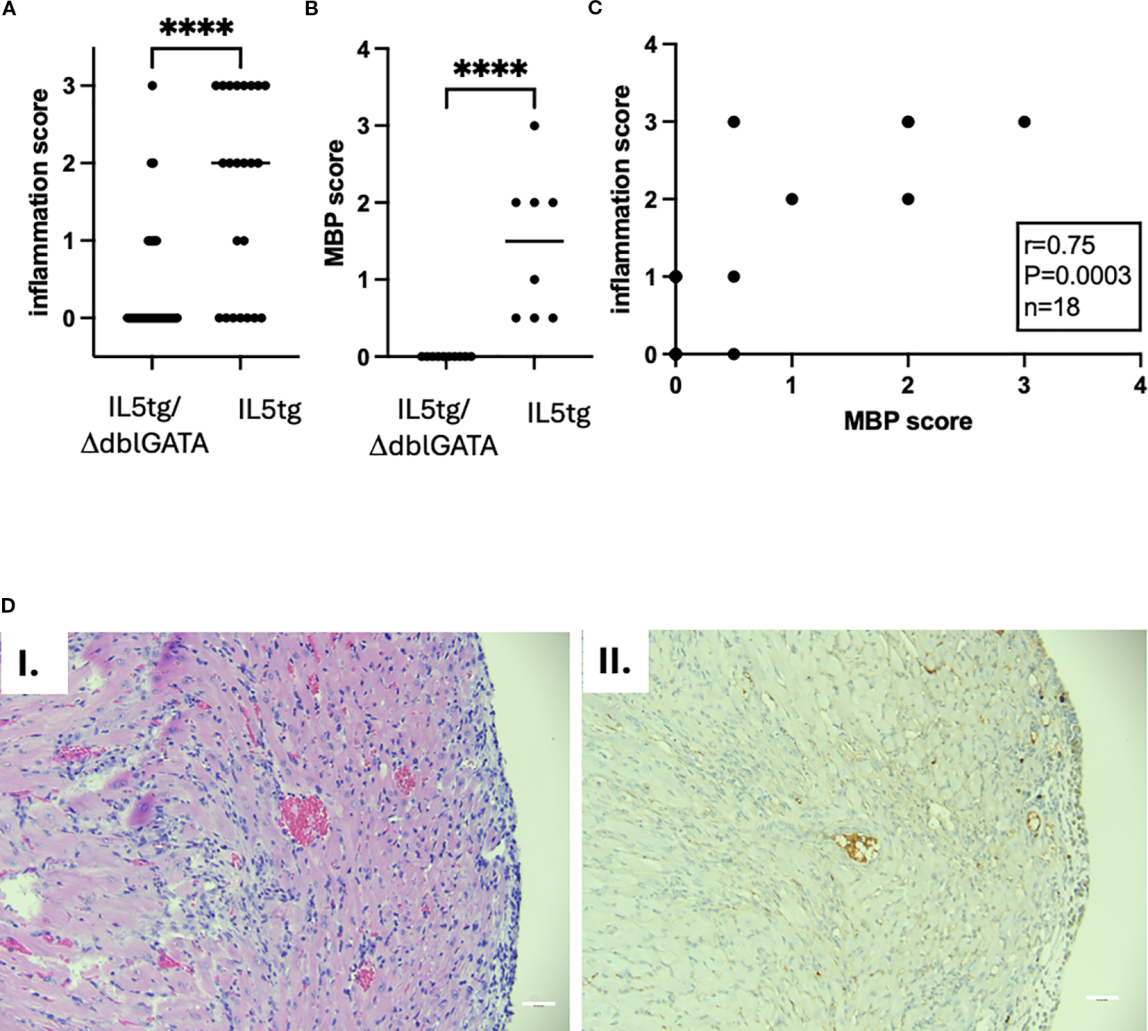
Heart inflammation in ΔdblGATA mice challenged with myocarditic peptide. **(A)** Heart inflammation was assessed by histology (semiquantitative assessment by observed blinded to treatment: 0 = none, 1 = mild, 2 = moderate, 3 = severe) from IL-5tg/ΔdblGATA (*n* = 33 mice) and IL-5tg mice (*n* = 23 mice, from five independent experiments). *p*-value by Mann–Whitney test; ****p < 0.0001. **(B)** MBP staining (denoting eosinophils) was quantified (semiquantitative assessment by observed blinded to treatment: 0 = none, 0.5 = scattered background eosinophils, 1 = mild infiltration, 2 = moderate infiltration, 3 = severe infiltration). Data shown are from 10 IL-5tg/ΔdblGATA and 8 IL-5tg mice from a representative experiment. *p*-value by Mann–Whitney test; ****p < 0.0001. **(C)** Correlation of heart inflammation assessed by histology and eosinophil inflammation assessed by MBP staining. Statistics were performed by Spearman correlation. **(D)** Histology of the IL-5tg/ΔdblGATA mouse with representative inflammation is shown with H&E (I) and MBP (II) staining. Magnification: 200×; scale bar, 50 mm.

The effect seen in IL-5tg mice can be due to the direct effect of IL-5, or via cells it activates including B cells and eosinophils. In order to test the role of eosinophils in heart inflammation, we used constitutively eosinophil-deficient mice (ΔdblGATA) challenged with the myocarditic peptide. Myocarditis penetrance was lower in ΔdblGATA (24% in ΔdblGATA/IL-5tg, compared with 74% in IL-5tg mice genetically matched and in the same experiments, Fisher’s exact test *p* = 0.0003). Furthermore, mice that did have heart inflammation had a lower intensity of inflammation (**Figure 3A**) and only rare eosinophils (as seen by H&E and MBP staining, **Figures 3B–D**) and no definitive cardiomyocyte damage. There was a significant correlation between MBP and inflammation scores (Spearman *r* = 0.75; *p* = 0.0003, **Figure 3C**). Thus, these data suggest that eosinophils are critical for heart inflammation in hypereosinophilia-associated heart disease.

### Single-cell RNA sequencing

**Figure 4:**
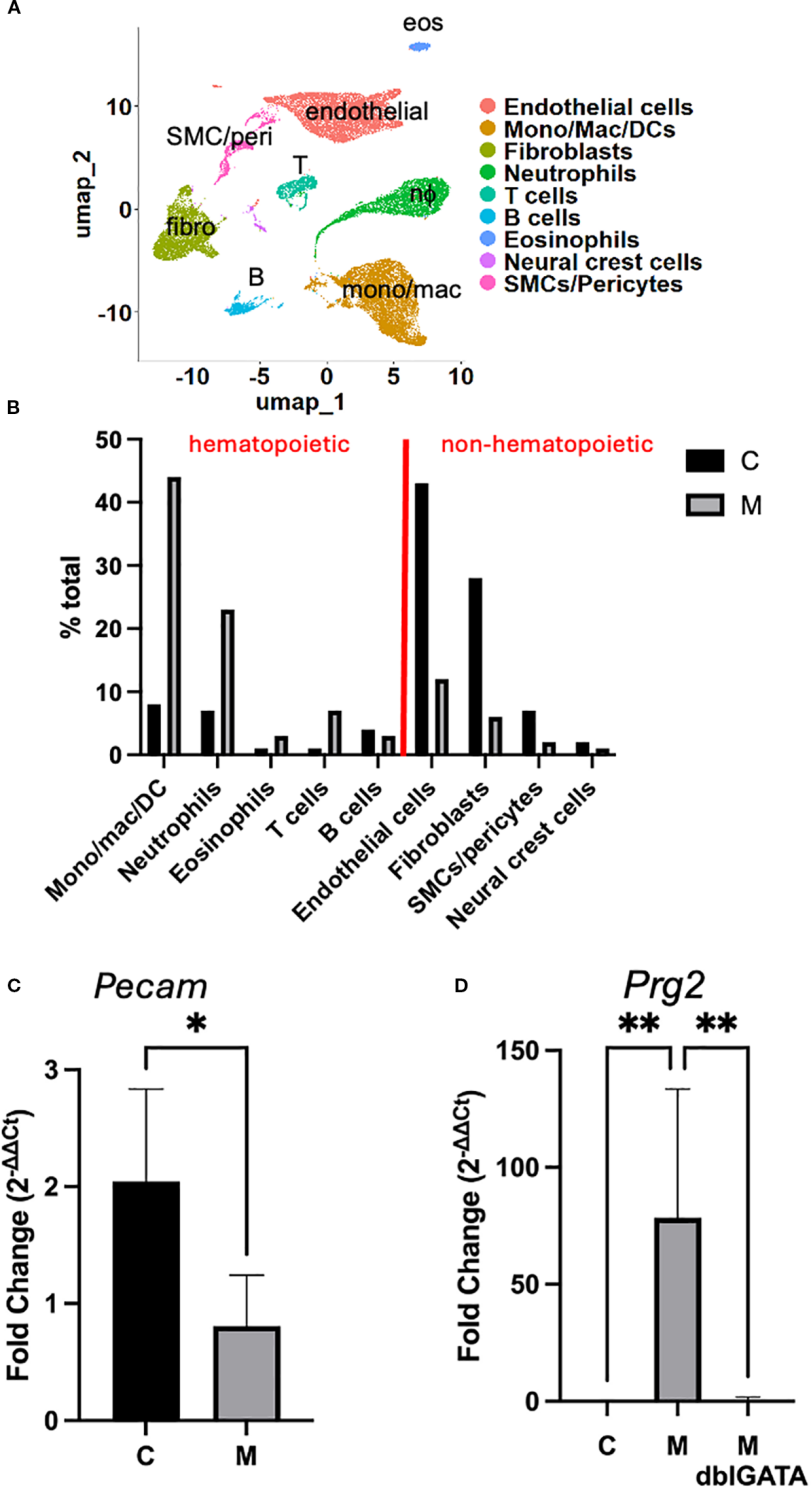
Cell clusters in eoEAM. **(A)** Cluster analysis of cells from the heart of myocarditic and control peptide challenged mice (integrated) is shown. **(B)** Proportion of cell types in the heart of myocarditic (M) and control **(C)** peptide challenged mice is shown. **(C)** *Pecam* gene expression was assessed by quantitative RT-PCR in hearts from mice challenged with myocarditic (M) or control (C) peptide from a representative experiment out of four is shown (*n* = 2 C mice and 7 M mice). **(D)** Expression of *Prg2* from a representative of two experiments is shown [*n* = 5 IL-5 transgenic mice each challenged with myocarditic (M) or control (C) peptide, and 5 IL-5 transgenic, eosinophil-deficient mice (ΔdblGATA) challenged with M peptide]. * P<0.05, ** P<0.01.

To interrogate cellular composition and diversity in their transcriptome profile, we performed scRNA-seq using hearts from mice challenged with the control (C) and myocarditic (M) peptide. Single-cell suspensions (16,000 cells/mouse) from hearts of challenged mice were loaded into the 10x Genomics chromium 3' expression system, and their libraries were sequenced for downstream analysis. A combined total of 25,885 cells from C (*n* = 11,770) and M (*n* = 14,115) hearts were analyzed using the Seurat R package and unbiased clustering yielded 13 clusters. Clusters were annotated using a combination of canonical markers (**Supplementary Table S1**), comparison to Immgen database, and FindAllMarkers function in Seurat; because of overlap of annotations for some of the clusters, the final annotation resulted in nine cell-type groups (**Figure 4A**). By comparing the relative proportions of each cell type in both treatment groups, we found that mono/mac/DCs, neutrophils, T cells, and eosinophils are enriched, B cells are not changed, and fibroblasts and endothelial cells are decreased in myocarditic compared with control hearts (**Figure 4B**). This is consistent with flow cytometry data (**Figure 1C**) that showed increased proportion of eosinophils and T cells. In order to verify findings generated by scRNA-seq by an independent method, we performed quantitative RT-PCR (qRT-PCR) for selected genes as markers of cell types whose amounts were changed. In **Figure 4C**, the expression of the *Pecam1* gene, used as a marker of endothelial cells, shows relatively decreased expression in mice challenged with myocarditic compared with control peptide, consistent with findings of scRNA-seq (**Figure 4B**). In contrast, expression of *Prg2*, a marker of eosinophils, was increased in mice challenged with the M peptide (**Figure 4D**). Importantly, the level of *Prg2* was not increased in ΔdblGATA mice challenged with the M peptide, consistent with their lack of eosinophils. Together, these data suggest that hematopoietic cells, especially mono/mac/DC, granulocytes, and T cells, have a significant role in the pathogenesis of hypereosinophilia-associated myocarditis.

**Figure 5:**
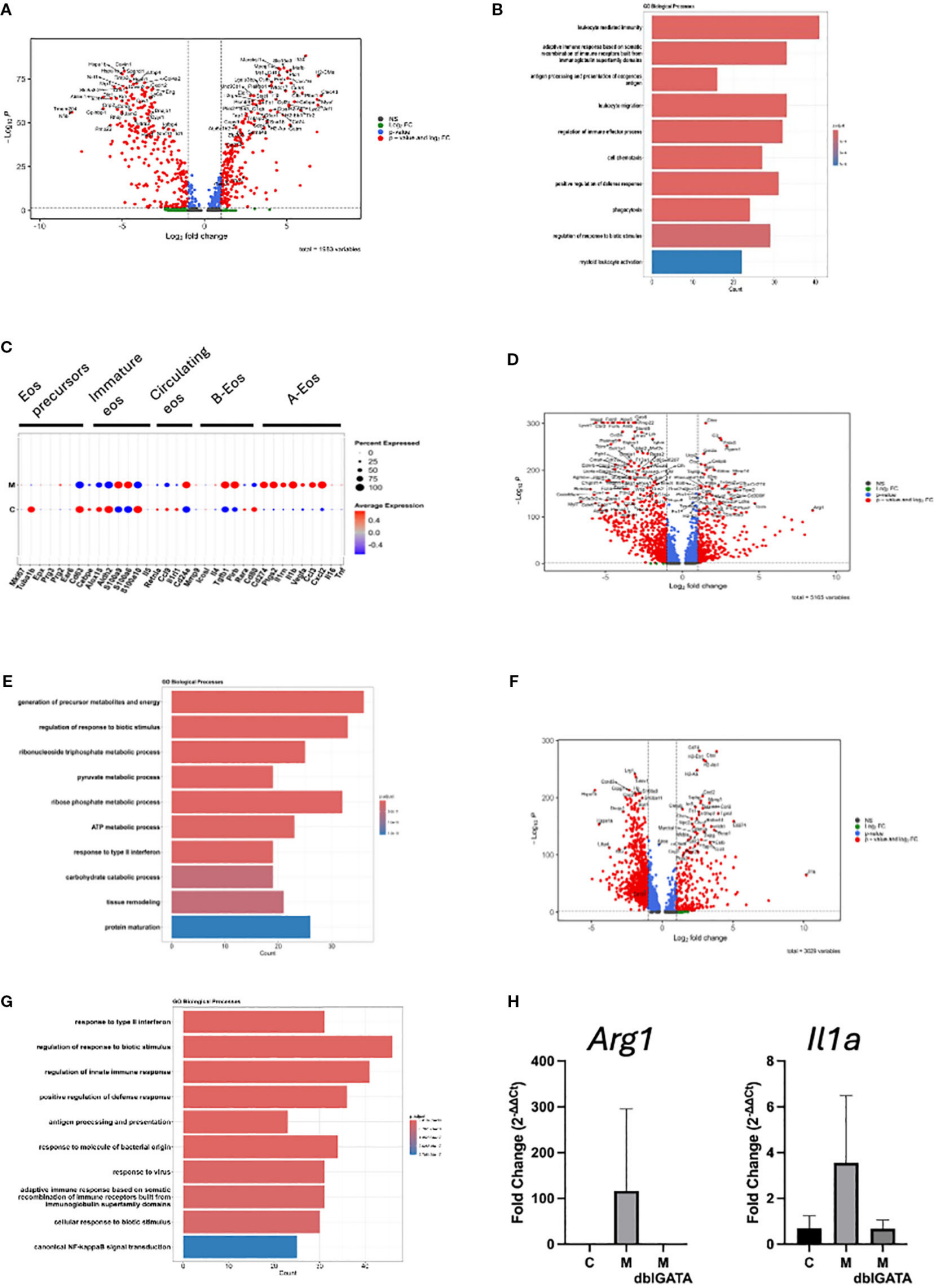
Differential gene expression in cell clusters. Differentially expressed genes in eosinophils are shown in the volcano plot **(A)** and the top 10 GO biological processes from upregulated genes **(B)**. **(C)** Scaled average expression and percent expression of cluster marker genes from Gurtner et al. (14) in eosinophils from M and C hearts are shown. Differentially expressed genes in mono/mac/DC are shown in the volcano plot **(D)** and the top 10 GO biological processes from upregulated genes **(E)**. Differentially expressed genes in neutrophils are shown in the volcano plot **(F)** and the top 10 GO biological processes from genes upregulated in M mice **(G)**. For volcano plots, genes with average log_2_ fold change >1 (Log_2_ FC), adjusted *p*-value <0.05 (*p*-value), or both (*p*-value and Log_2_ FC) were used. **(H)** Expression of *Arg1* and *IL1ra* from a representative of three experiments is shown [*n* = 5 IL-5 transgenic mice each challenged with myocarditic (M) or control (C) peptide, and 5 IL-5 transgenic, eosinophil-deficient mice (ΔdblGATA) challenged with M peptide].

To determine the functional roles of the enriched cell types, we compared gene expression profiles between the two treatment groups. We first focused on eosinophils because experiments in eosinophil-deficient mice demonstrated their important role in heart inflammation. We found 231 significantly upregulated and 269 significantly downregulated genes in eosinophils from the heart of mice challenged with myocarditic peptide compared with control peptide (red labeled genes in the volcano plot in **Figure 5A**). We next assessed for biological processes associated with the upregulated genes in eosinophils and found an increase in the expression of genes associated with regulation of immune effector functions including phagocytosis and other defense responses, leukocyte migration/chemotaxis, and antigen processing and presentation (**Figure 5B**). In order to assess the subtype/activation state of eosinophils in C and M hearts, we assessed expression of marker genes from Gurtner et al. (14). The pattern of genes with increased expression in M hearts (including *Cd274/PDL1, Ptgs2, Il1rn, Il1b, Vegfa, Ccl3*, and *Cxcl2*) is consistent with a type 1 polarized (28) or activated (A) (14) phenotype (**Figure 5C**).

Next, we turned to monocytes/macrophages/DCs, which were the most abundant cells in hearts from mice challenged with myocarditic mice. We found 355 upregulated and 652 downregulated genes in mono/mac/DC from the heart of mice challenged with the myocarditic peptide compared with the control peptide (**Figure 5D**). A review of enriched biological processes identified genes involved in glycolysis, urea cycle, cellular response to hypoxia, and cell death processes as enriched (**Figure 5E**). Finally, we analyzed DEGs in neutrophils. We found 260 upregulated and 932 downregulated genes in neutrophils from heart of mice challenged with the myocarditic peptide compared with the control peptide (**Figure 5F**). A review of enriched biological processes identified genes involved in cellular response to LPS, inflammasome-mediated signaling, antigen processing and presentation, and cell death processes as enriched (**Figure 5G**). Top upregulated genes in mono/mac/DC and neutrophils, *Arg1* and *Il1a*, respectively, were confirmed by qRT-PCR (**Figure 5H**). While their increase was not statistically significant in this particular experiment, they were consistently increased over three experiments in mice with histologically proven inflammation (data not shown). Importantly, there was no increase in *Arg1* or *Il1a* in eosinophil-deficient mice (**Figure 5H**), suggesting that eosinophils are required for activation of mono/macs and neutrophils.

**Figure 6:**
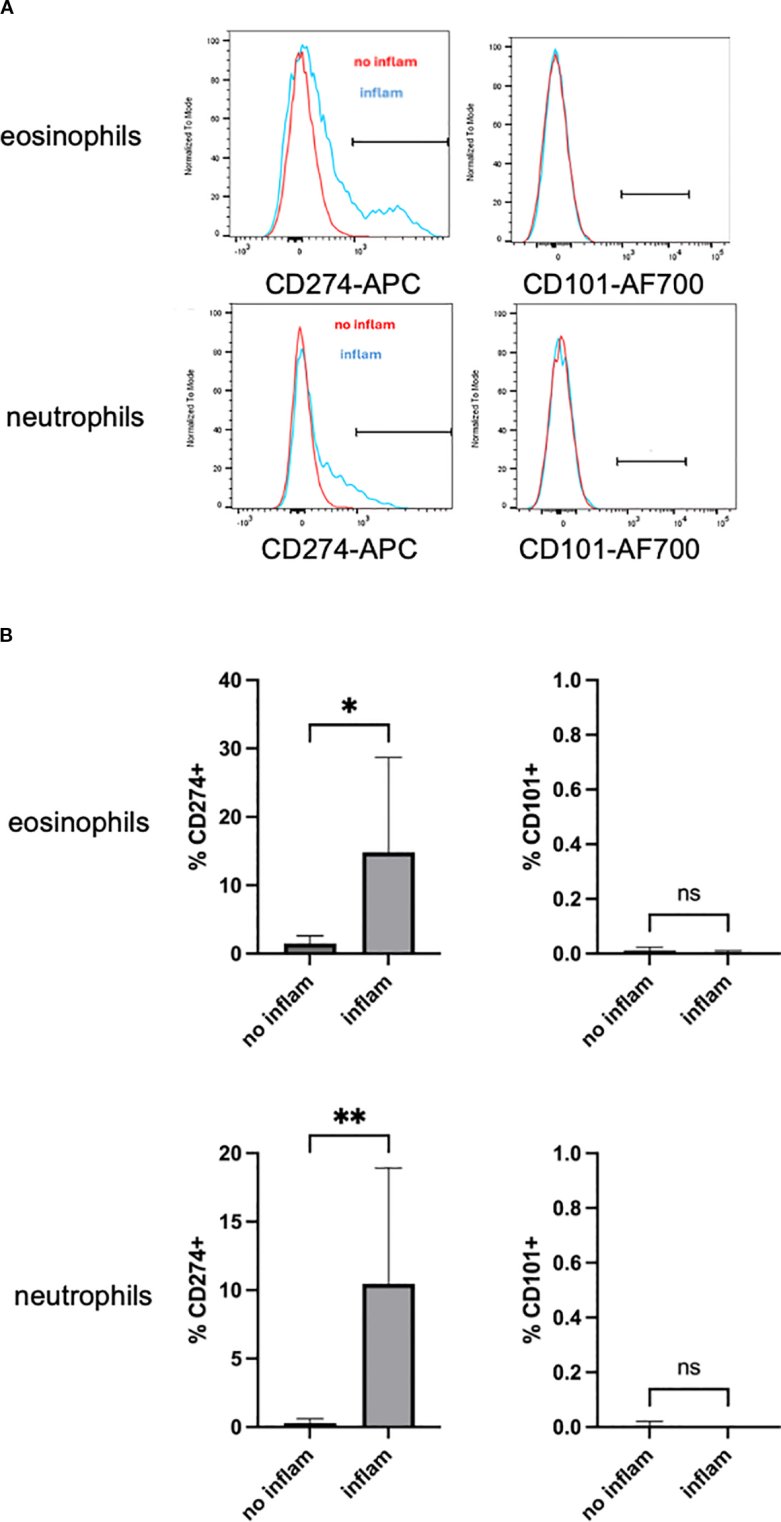
Assessing cell activation status in eoEAM. Representative histogram overlays showing surface protein expression of CD274 and CD101 on eosinophils and neutrophils from mice with (inflam, defined as inflammation score ≥1) and without (no inflam, defined as inflammation score = 0) histological inflammation in the heart are shown in **(A)** Comparison on mice with (inflam, *n* = 4) and without (no inflam, *n* = 7) ventricular inflammation by histology from a representative of three total experiments is shown in **(B)** *p*-value by unpaired *t*-test. Ns = not significant, * *p* < 0.05, ** *p* < 0.01.

To verify findings generated by scRNA-seq on a protein level, we performed flow cytometry analysis and assessed the level of CD274 and CD101 on eosinophils and neutrophils in the heart of eoEAM mice, since CD274 and CD101 are biomarkers of type 1 and type 2 polarized eosinophils, respectively (28), and both are present on subsets of neutrophils (29, 30). The proportion of cells expressing surface CD274 increased on both neutrophils and eosinophils, particularly in mice that showed inflammation by histology (**Figure 6**). Importantly, the proportion of CD274-expressing cells was not different in the spleen of challenged mice, irrespective of heart inflammation (data not shown). There was no significant increase in expression of CD101 on either eosinophils or neutrophils in the heart (**Figure 6**). There was no difference in the levels of CD274-positive eosinophils or neutrophils between unchallenged mice, mice challenged with the C peptide, or those challenged with the M peptide that did not show inflammation by histology (data not shown).

### *In vitro* activation of eosinophils

**Figure 7:**
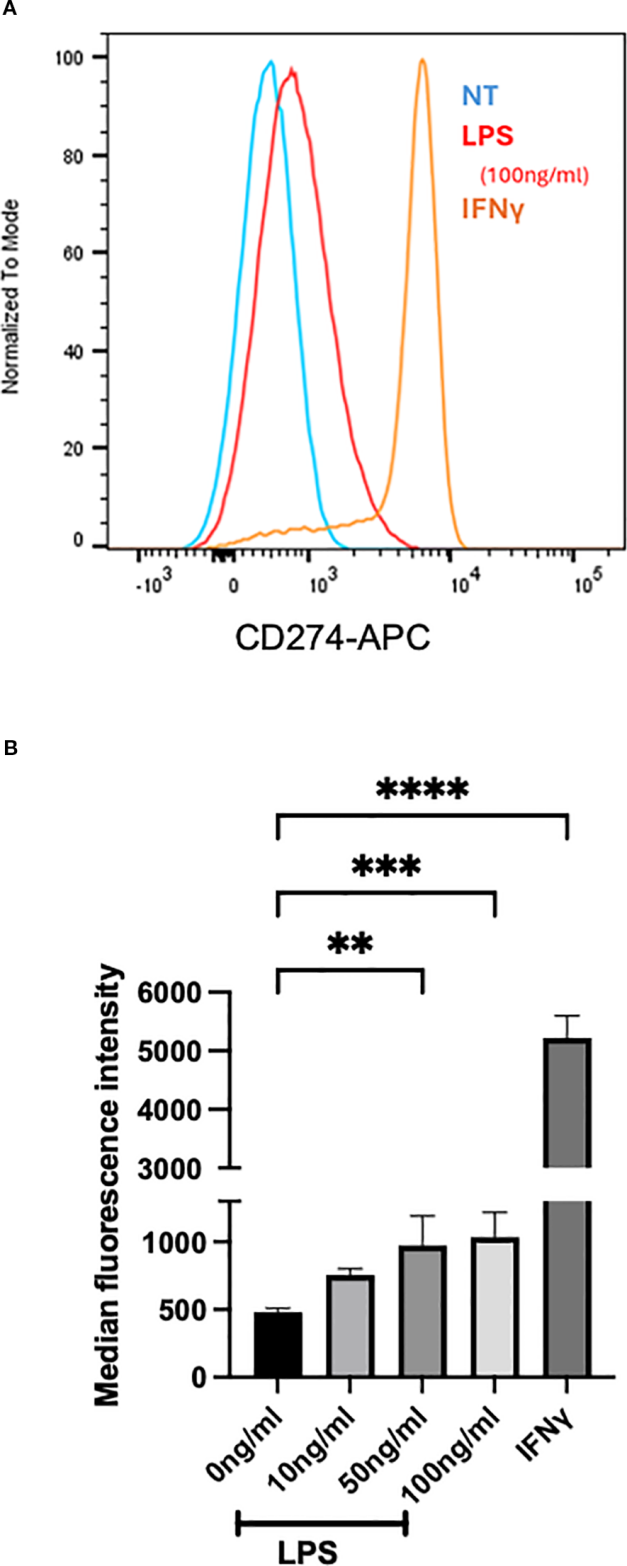
*In vitro* stimulation of BMDeos. **(A)** Representative histogram overlays show surface expression of CD274 on BMDeos following stimulation. **(B)** Quantification of CD274 surface expression (median fluorescence intensity, MFI) after 18-h stimulation with LPS (10, 50, and 100 ng/mL) and IFNγ (50 ng/mL) is shown. Statistical analysis was performed using one-way ANOVA followed by Dunnett’s multiple comparisons test. Data are presented as mean ± SD of *n* = 4 individual experiments. Significance levels are indicated as **p < 0.01, ***p < 0.001, and ****p < 0.0001.

Having seen the subset of heart eosinophils in inflamed mice having a type 1 phenotype (28), we assessed whether eosinophils can be similarly activated *in vitro*. We assessed whether BMDeos can be stimulated to express CD274, a marker of type 1 eosinophils (28). Eosinophils were stimulated with multiple stimuli, including cytokine IFNγ (previously shown inducer of type 1 polarized eosinophils) and LPS (a pathogen-associated molecular pattern found in adjuvant we used *in vivo*, chosen because our scRNA-seq data showed that pattern recognition receptors were upregulated on eosinophils). IFNγ markedly increased the expression of CD274 (**Figure 7**), consistent with type 1 polarization. Furthermore, BMDeos stimulated with LPS showed a concentration-dependent increase in the level of CD274 expression (**Figure 7**). In summary, stimulation of eosinophils with specific stimuli *in vitro* recapitulates type 1 activation state seen *in vivo*.

## Discussion

In this manuscript, we used a model of hypereosinophilia-associated heart disease to investigate the molecular and cellular mechanisms of disease.

As the NIH TREAD and recent RE-TREAD reported, there is a paucity of preclinical models that adequately replicate cardiac disease in hypereosinophilia, and development of these models would enable mechanistic studies aiming to develop targeted therapies (8, 9). Attempts to model hypereosinophilia-associated heart disease in mice include spontaneous eosinophilic myocarditis in DBA/2 (D2) mice (31), baseline heart disease in aging IL-5tg mice (32), and a model of antigen (cardiac myosin)-induced autoimmune EM elicited in IL-5tg mice (12, 33). The D2 model is limited in that it lacks the endocardial thrombosis, fibrosis, and systemic aspects typical of HES; furthermore, this model develops very early and resolves on its own by ~3 months of age in contrast to progressive disease seen in patients (34). Diny et al. have shown that IL-5tg mice develop worsening left ventricular function with age; however, no thrombosis or fibrosis was seen (32). Studies in myosin peptide-challenged IL-5tg mice developed severe inflammation, followed by DCM and fibrosis (12). It is important to note here that Diny et al. used mice where the IL-5 transgene is driven by the CD3 promoter (35), while our studies use mice where the IL-5 transgene is driven by the CD2 promoter (15). While IL-5 is produced by T cells in both lines, the level of eosinophilia differs between the two mouse lines, with CD3.IL-5tg mice having higher baseline circulating eosinophil levels than CD2.IL-5tg (40%–60% versus 20%–30%, respectively). Thus, findings from one line cannot be directly assumed to translate to the other line.

Eosinophils have been shown to play both host protective and destructive roles in different models, and the mechanism of their involvement involves multiple effector functions including contributing to antigen presentation and modulation of adaptive immune responses; damage to tissues by cytotoxic granule proteins or antibody-dependent cellular cytotoxicity; and promoting inflammation, thrombosis, and/or tissue repair and angiogenesis via the secretion of cytokines, chemokines, and tissue factor (36, 37). For instance, eosinophils and/or their granule proteins are cytotoxic in a variety of scenarios, including data in cardiomyocytes (38, 39). In cardiovascular models, eosinophils have also shown both protective and destructive roles. For instance, in models of myocardial infarction and hypertrophic injury, eosinophils are cardioprotective via the production of IL-4 (40–42). However, in models of myocarditis (not associated with hypereosinophilia) and atherosclerosis, they were pathogenic as shown by improved outcomes in eosinophil-deficient mice (12, 43). Thus, it was critical to test whether eosinophils are protective or detrimental in hypereosinophilia-associated heart disease. Our studies show that eosinophils are critical for inflammation in that both the occurrence and severity of inflammation are decreased in ΔdblGATA eosinophil-deficient mice. Future studies will examine the specific mechanism of eosinophil-mediated pathogenesis in hypereosinophilia-associated heart disease, such as a hypothesized role via direct cytotoxicity to cardiomyocytes by eosinophil granule proteins, or promoting inflammation via cytokines and/or chemokines. In summary, consistent with previous findings in other organs, the role of eosinophils in the heart depends on the nature of the inflammatory response.

In order to study the mechanism, we performed scRNA-seq analysis from hearts of mice challenged with the myocarditic or control peptide. We focused on the eosinophil phenotype as our data in eosinophil-deficient mice showed that eosinophils are pro-inflammatory and important for the development and severity of heart inflammation. Previous studies using scRNA-seq found eosinophils to be difficult to assess with flow-based methods (such as 10x Genomics); gravity-based scRNA sequencing approaches (such as BD rhapsody) appeared to be more successful (14, 44). Considering the partial penetrance of disease and thus not knowing up front which mice had developed heart inflammation, we fixed and froze the heart single-cell suspensions, and performed scRNA-seq with previously fixed cells once we knew which mice had developed the disease. We found that there was increased yield of eosinophils compared with historic experience with flow-based scRNA-seq methods, albeit eosinophil yield was lower than seen by other methods such as flow cytometry and histology. Thus, using fixed cells with flow-based methods can be beneficial for scRNA-seq assessment of granulocytes.

Previous studies have shown eosinophil heterogeneity between and within organs in homeostasis (13, 14). Furthermore, with *in vitro* activation and in different disease models, there is expansion of eosinophils in different activation states (14, 28). Specifically, type 1 eosinophils are activated *in vitro* with IFNγ and/or bacterial products, are present in the gastrointestinal tract at baseline, and expand with models of bacterial infection or chemical colitis (14, 28). In contrast, type 2 eosinophils are activated *in vitro* by IL-4, and are prominent in the lung in models of allergic inflammation (28, 45). Based on the transcriptional profile and surface assessment of CD274 and CD101, our data show that eosinophils in the heart of hypereosinophilia-associated myocarditis are type 1 polarized. Type 1 eosinophils have been shown to regulate host defense and immune responses in other diseases, and our study now supports their role in heart inflammation. However, while they served to prevent excessive tissue damage in colitis (14), in models of lung inflammation, CD274-positive eosinophils promoted inflammation (46). Thus, the role of CD274-expressing eosinophils in the model of heart inflammation will need to be directly tested in future studies. Future comparative studies will aim to understand the differing mechanisms between inflammation in different organs. In humans, a very small population of peripheral blood eosinophils express CD274, but this increases in the nasal mucosa (47). To the best of our knowledge, expression of PDL1 on eosinophils has not been tested in myocarditis. Published studies in myocarditis (not related to hypereosinophilia) have shown the PD1/PDL1 pathway to have a cardioprotective role. For instance, myocarditis is a rare but potentially fatal immune-related adverse event in patients treated with immune checkpoint inhibitors such as antibodies against PD1 and/or PDL1 (48). Mouse models of myocarditis, stress-induced cardiomyopathy, and neonatal heart injury show the protective role of the PD1/PDL1 axis and involve PDL1 expression on macrophages, dendritic cells, and endothelial cells (49–51). Owing to the paucity of data, future studies will focus on assessing the expression of PDL1 in biopsies from patients with hypereosinophilia-associated heart disease, as well as potential mechanisms for its regulation and function. Importantly, eosinophils have both immunoregulatory and pathogenic roles and there is inter- and intra-tissue heterogeneity; thus, increased understanding of different subsets/activation states will improve our ability to target therapies specifically to the pathogenic ones while sparing beneficial eosinophils, and will enable us to consider specific eosinophil subsets as diagnostic or prognostic biomarkers.

Both scRNA-seq and flow cytometry showed increased eosinophils in inflamed hearts. However, eosinophils were detectable in control peptide challenged mice, where they are not seen by H&E histology. Considering both scRNA-seq and flow cytometry are performed on single-cell suspensions from the organ, these cells are most likely derived from circulation (hearts were flushed, but there are remaining white blood cells in heart blood vessels). In mice challenged with myocarditic peptide and with tissue inflammation, while some eosinophils may also be circulation derived, majority are likely tissue invading, as supported by histologic assessment. This is also supported by the finding that CD274 is present only on a subset of eosinophils from inflamed hearts (**Figure 6**) and the finding that the proportion of CD274-expressing cells was not different in the spleen of challenged mice, irrespective of heart inflammation (data not shown). Future studies should use spatial approaches, such as spatial transcriptomics or dual immunohistochemical staining with eosinophil markers and CD274, to directly test this hypothesis.

The presence of different subsets/activation states of eosinophils in organs at times of inflammation may be due to the preferential recruitment of specific subsets or their differentiation *in situ*. We performed *in vitro* experiments where BMDeos were stimulated with various stimuli present in the microenvironment. As seen in **Figure 7**, expression of CD274, a biomarker of type 1 eosinophils, can be induced by specific stimuli. These data support the notion that the local inflammatory milieu is responsible for eosinophil activation. Furthermore, in our model, both the control and myocarditic peptides are emulsified in mycobacteria-containing adjuvant. However, even though both groups received systemic adjuvant and had increased innate immunity (e.g., see **Figure 1B**), only eosinophils in the heart (as opposed to spleen) and only the ones that had heart inflammation had increased CD274 expression (**Figure 6**). This finding further supports the hypothesis that the local inflammatory milieu is responsible for eosinophil activation.

In summary, eosinophils are required for heart damage in hypereosinophilia-associated heart disease. Furthermore, eosinophils in the heart of hypereosinophilia-associated heart disease are type 1 activated and may contribute to disease pathogenesis.

## Data Availability

The datasets presented in this study can be found in online repositories. The names of the repository/repositories and accession number(s) can be found in the article/**Supplementary Material**.
