# Supplementary material for: The role of eosinophils and their activation state in hypereosinophilia-associated heart disease
Source: Front Immunol. 2025 Sep 19;16:1635483. doi: 10.3389/fimmu.2025.1635483 (PMC12491288; doi:10.3389/fimmu.2025.1635483)
Supplement: Supplementary file 1 [file Table1.pdf]

Table S1: Marker genes of cell types

Cell Type	Marker Genes
Eosinophils	*Epx, Prg2, Siglecf, Ccr3, Il5ra*
Neutrophils	*Csf3r, S100a8, S100a9*
Mast cells	*Fcer1a, Mcpt4, Tpsb2, Cpa3*
Monocytes & Macrophages	*Cd68, Cd163*
Dendritic cells	*Cd209a, Cd207, Fit3*
B cells	*Jchain, Ighm, Igkc*
T cells	*Cd3e, Cd4, Icos*
Endothelial cells	*Pecam1, Esam, Selp*
Fibroblasts	*Postn, Fbln1, Fbln2*
Mesothelial cells	*Upk3b, Msln*
Sensory neurons	*Piezo2, Grm5*
Epithelial cells	*Krt4, Krt13*
