# Supplementary material for: The role of eosinophils and their activation state in hypereosinophilia-associated heart disease
Source: Front Immunol. 2025 Sep 19;16:1635483. doi: 10.3389/fimmu.2025.1635483 (PMC12491288; doi:10.3389/fimmu.2025.1635483)
Supplement: Supplementary file 3 [file Presentation1.pdf]

Supplementary methods: flow cytometry compensation and gating strategy

Minimum Information about a Flow Cytometry Experiment (MIFlowCyt)

A. **Eosinophilic experimental autoimmune myocarditis (eoEAM) experiment**
B. **Assessment of surface markers after stimulation of bone marrow derived eosinophils (BMDeos)**

1. Experiment Overview

1.1. Purpose

A. The purpose of the experiment is to quantify the proportion and determine the activation status of infiltrating hematopoietic cells by flow cytometric analysis in hypereosinophilia- associated heart disease of eosinophilic experimental autoimmune myocarditis (eoEAM) mouse model.
B. The purpose of the *in vitro* experiment was to define the activation phenotype of bone marrow derived eosinophils (BMDeos) stimulated with various stimuli.

1.2. Keywords

Heart, spleen, cardiac cell, splenic cell, T cell, B-cell, eosinophil, neutrophil

1.3. Experiment Variables

A. Induction of myocarditis: Mice were stimulated with myocarditic versus control peptide. Flow cytometry was used to identify cell types and their activation state.
B. Stimulation with cytokines and pathogen-associated molecular patterns (PAMP): BMDeos were stimulated in vitro with cytokines and PAMPs, and their surface expression of activation markers.

1.4. Organization

1.4.1. Name: University of Cincinnati College of Medicine, Department of Pathology and Laboratory Medicine
1.4.2. Address: 3230 Eden Ave, Cincinnati, OH 45267; USA

1.5. Primary Contact

1.5.1. Name: Nives Zimmermann
1.5.2. Email: zimmerns@ucmail.uc.edu

1.6. Date

A. Multiple *in vivo* experiments were performed between December 2022 and November 2024. Flow cytometry analysis performed 3 weeks post injection.

B. Multiple *in vitro* experiments were performed between November 2024 and May 2025.

1.7. Conclusions: see manuscript.

1.8. Quality Control Measures
A. Splenic cells collected from challenged mice were used in order to provide staining controls such as fluorescence minus one (FMO, for gating), and single stained controls (for compensation). Cardiac and splenic cells were used for unstained control.
B. Unstimulated and stimulated BMDeos were used for compensation controls, gating and FMO.

1.9. Other Relevant Experiment Information: N/A

2. Flow Sample/Specimen Details

2.1. Sample/Specimen Material Description

2.1.1. Biological Samples
2.1.1.1. Biological Sample Description: Spleen and heart single cell suspensions.
2.1.1.2. Biological Sample Source Description: Mus musculus, BALB/c background, CD2.IL5 transgenic
2.1.1.3. Biological Sample Source Organism Description:
A. In vivo experiments

- *Taxonomy*: Mus musculus, BALB/c background
- *Age*: >6wk-old mice
- *Gender*: Female and male
- *Phenotype*: white, hypereosinophilic
- *Genotype*: CD2.IL5 transgenic mice (CD2.IL5tg)
- *Treatment*:
    - 100 µg myosin heavy chain α (MyHCα) 614 peptide (Ac-SLKL MATL FSTY ASAD; Genscript) for M- group,
    - or 790 peptide (Ac-IQAQ ARGQ LMRI EFKK) for C-group
    - Unchallenged group
- *Irradiation*: N/A
- *Other Relevant Biological Sample Source Organism Information:* The animal care and house for use at the University of Cincinnati (UC) under Laboratory Animal Medical Services (LAMS).

B. In vitro experiments

- *Taxonomy*: Mus musculus , BALB/c background
- *Age*: >6wk-old mice
- *Gender*: Female
- *Phenotype*: white
- *Genotype*: wild type
- *Stimulation:*
    - No *in vivo* treatment

- BMDeos with LPS (10, 50, 100ng/ml), IL-5 (10ng/ml), PolyIC (10ug/ml), TNFα (20ng/ml), protein A/G (2ug/ml), CD95 (100ng/ml) and CD95 crosslinked with protein A/G (cCD95)

2.1.1.4. Other Relevant Biological Sample Information:
Mouse ID was used as sample/file identifier.
2.1.2. Environmental Samples: N/A
2.1.3. Other Samples: N/A

2.2. Sample Characteristics

A. Expected/analyzed types of cells: cardiac and splenic single cell suspensions are stained for hematopoietic cells and their subtypes (lymphocytes, granulocytes).
B. BMDeos are a pure cell population.

2.3. Sample Treatment Description

A.
- Single cell suspensions were prepared (as described in material and methods of main manuscript). Cell concentration was adjusted to 10^6/200ul.
- Fc receptors were blocked using anti-mouse CD16/CD32 antibody (Invitrogen eBioscience) for 10 minutes on ice in FACS buffer. The cells are not washed before the first staining step.
- Cells were incubated with antibodies (or controls) for 30 minutes at 4°C in staining buffer (approx. 10^6 cells in 200µl of staining buffer).
- After the incubation, cells were washed 1x with 1ml of staining buffer and pelleted by centrifugation (400g for 5 min); supernatant has been removed.
- Finally, cells were resuspended in 400ul FACS buffer with 7AAD (bioscience ref# 006993-50) and incubated at room temperature for 15min prior to flow analysis

B.
- After treatment, BMDeos were collected and washed with FACS buffer
- Fc receptors were blocked using anti-mouse CD16/CD32 antibody (Invitrogen eBioscience) for 10 minutes on ice in FACS buffer. The cells are not washed before the first staining step.
- Cells were incubated with antibodies (or controls) for 30 minutes at 4°C in staining buffer (approx. 10^6 cells in 200µl of staining buffer).
- After the incubation, cells were washed 1x with 1ml of FACS buffer and pelleted by centrifugation (300g for 5 min); supernatant has been removed.
- Finally, cells were resuspended in 400ul FACS buffer with 7AAD (bioscience ref# 006993-50) and incubated at room temperature for 15min prior to flow analysis

2.4. Fluorescence Reagent Description

Compensation tubes have been created as follows:

Reporter	PE	APC	FITC	BV421	PEcy7	APCcy7	BV605	7AAD	none
Tube#1	+Ab	-	-	-	-	-	-	-	-
Tube#2	-	+Ab	-	-	-	-	-	-	-
Tube#3	-	-	+Ab	-	-	-	-	-	-
Tube#4	-	-	-	+Ab	-	-	-	-	-
Tube#5	-	-	-	-	+Ab	-	-	-	-
Tube#6	-	-	-	-	-	+Ab	-	-	-
Tube#7	-	-	-	-	-	-	+Ab	-	-
Tube#8	-	-	-	-	-	-	-	7AAD	-
Tube#9	-	-	-	-	-	-	-	-	-

Each sample has been stained as follows:

Reporter	PE	APC	FITC	BV421	PEcy7	APCcy7	BV605	7AAD
Sample	+Ab	+Ab	+Ab	+Ab	+Ab	+Ab	+Ab	7AAD

The following reagents are used:

Immunophenotype

Analyte	Reporter	Detector	Target	Manufacturer	Cat#
Siglec-F	PE	CD170 (Siglec F) Antibody	eosinophils	Invitrogen	155506
CD19	APC	Anti-Mouse CD19 Antibody	B-lymphocytes	, Invitrogen	17019382
CD3	FITC	Anti-Mouse CD3e Antibody	T-lymphocytes	Invitrogen	11003182
Ly6G	BV421	Rat Anti-Mouse Ly-6G Antibody	Neutrophils	BD Horizon	562737
F4/80	PEcy7	anti-mouse F4/80 Antibody	Monocytes and macrophages	Biolegend	123113
viability	7AAD		Live/dead	eBioscience	006993-50

CD45	APCcy7	Rat Anti-Mouse CD45 Antibody	Hematopoietic cells	BD Pharmingen	1046748
CD49b	BV605	Anti-Mouse CD49b	NK cells	BD	569508
CD122	BV605	Rat anti-Mouse	NK cells	BD Biosciences	BDB745171

Activation status *in vivo*

**Analyte**	**Reporter**	**Detector**	**Target**	**Manufacturer**	**Cat#**
Siglec-F	PE	CD170 (Siglec F) Antibody	eosinophil	Invitrogen	155506
Ly6G	BV421	Rat Anti-Mouse Ly-6G Antibody	Neutrophil	BD Horizon	562737
viability	7AAD		Live/dead	eBioscience	006993-50
CD45	APCcy7	Rat Anti-Mouse CD45 Antibody	Hematopoietic cells	BD Pharmingen	1046748
CD274	APC	Anti-mouse CD274 (B7-H1, PD-L1) Antibody	Cell activation	Biolegend	cat#124311
CD101	Alexa Fluor700	Anti-mouse CD101 Monoclonal Antibody	Cell activation	eBioscience	Catalog # 56-1011-82

Activation status *in vitro*

**Analyte**	**Reporter**	**Detector**	**Target**	**Manufacturer**	**Cat#**
Siglec-F	PE	CD170 (Siglec F) Antibody	eosinophil	Invitrogen	155506
CCR3	BV421	anti-mouse CD193	eosinophil	Biolegend	cat# 144517

		(CCR3) Antibody			
viability	7AAD		Live/dead	eBioscience	006993-50
CD274	APC	Anti-mouse CD274 (B7-H1, PD-L1) Antibody	Cell activation	Biolegend	cat#124311
CD101	Alexa Fluor700	Anti-mouse CD101 Monoclonal Antibody	Cell activation	eBioscience	Catalog # 56-1011-82

3. Instrument Details

3.1. Instrument Manufacturer

Beckton Dickinson

3.2. Instrument Model

BD/FACSCanto
BD/LSRFortessa

Technical specification at:
https://www.cincinnatichildrens.org/research/cores/flow-cytometry

3.3. Instrument Configuration and Settings

3.3.1. Flow Cell and Fluidics

The instruments have not been altered

3.3.2. Light Sources

The instruments have not been altered

3.3.3. Excitation Optics Configuration

The instruments have not been altered.

Number of Fluorescence PMTs

Instrument Name*	Instrument Make/Model**	UV (355nm)	Violet (405nm)	Blue (488nm)	Yellow-Green (561nm)	Red (635nm)
**Canto 3**	BD/FACSCanto		3	5		3
**Instrument Name***	**Instrument Make/Model****	**UV (355 nm) 20mW**	**Violet (405 nm) 50mW**	**Blue (488 nm) 60mW)**	**Yellow-Green (561 nm) 50mW**	**Red (640 nm) 40mW**
**Fortessa**	BD/LSRFortessa	2	6	2	5	3

3.3.4. Optical Filters

The instruments have not been altered, all filters are original and came with the instrument.

3.3.5. Optical Detectors

The instruments have not been altered.

3.3.6. Optical Paths

The instruments have not been altered. The following figure shows the filter and detector configuration:

Optical detectors configuration_Cantos:

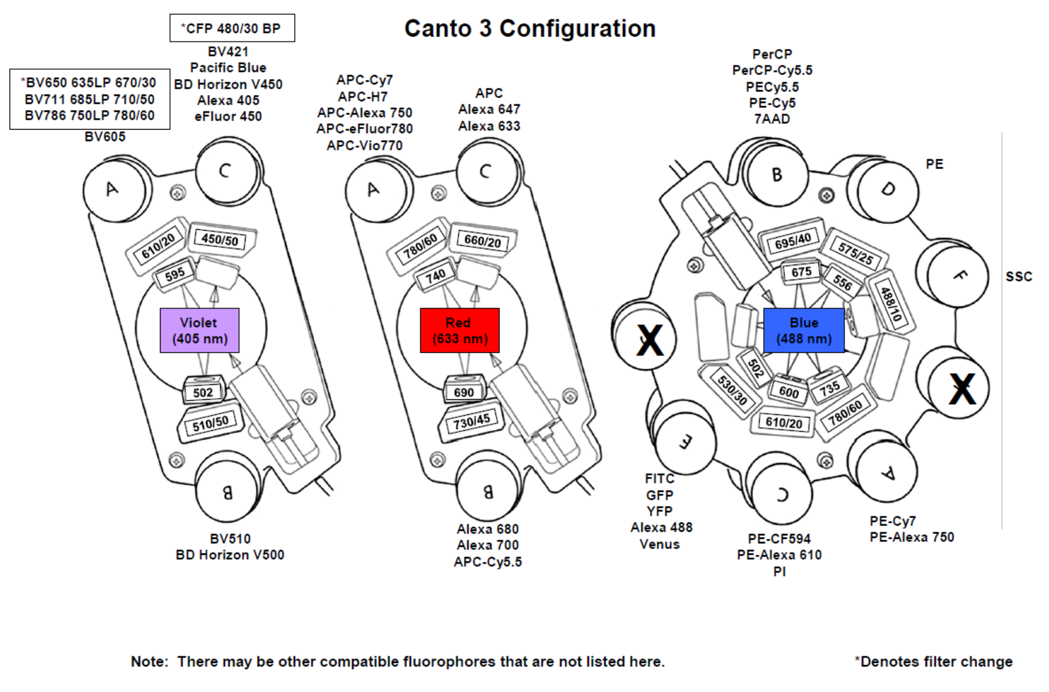

Optical detectors configuration detail_Cantos:

Detector Array (laser)	PMT	LP Mirror	BP filter	Dye/detector
Violet (405nm)	A	595	610/20	635LP 670/30<br>BV711 685LP 710/50<br>BV786 750LP 780/60<br>BV605
	B	502	510/50	BV510<br>BD Horizon V500
	C	-	450/50	BV421<br>Pacific Blue<br>BD Horizon V450<br>Alexa 405<br>eFluor 450<br>*CFP 480/30 BP
Red (633 nm)	A	740	780/60	APC-Cy7<br>APC-H7

				APC-Alexa 750<br>APC-eFluor780<br>APC-Vio770
	B	690	730/45	Alexa 680<br>Alexa 700<br>APC-Cy5.5
	C	-	660/20	APC<br>Alexa 647<br>Alexa 633
Blue (488nm)	A	735	780/60	PE-Cy7<br>PE-Alexa 750
	B	675	695/40	PerCP<br>PerCP-Cy5.5<br>PECy5.5<br>PE-Cy5<br>7AAD
	C	600	610/20	PE-CF594<br>PE-Alexa 610<br>PI
	D	556	575/25	PE
	E	502	530/30	FITC<br>GFP<br>YFP<br>Alexa 488<br>Venus
	F	-	488/10	

Optical detectors configuration_fortessa:

Fortessa 3 Configuration

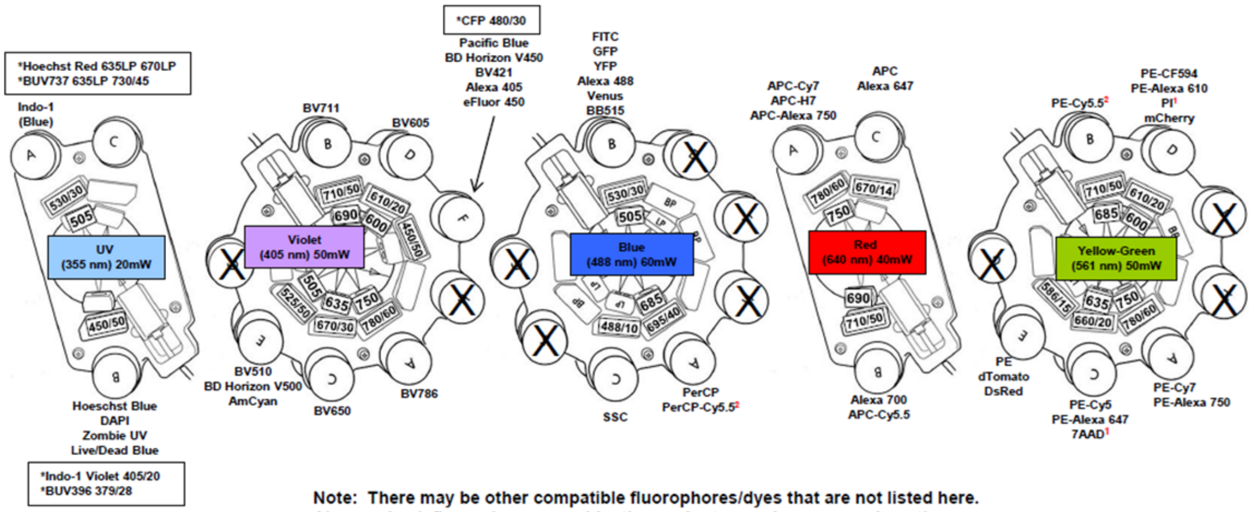

Note: There may be other compatible fluorophores/dyes that are not listed here.
Always check fluorochrome combinations prior to running an experiment!

***Denotes filter change**
[1]7AAD and PI may be problematic depending on your fluorochrome panel.
[2]PerCP-Cy5.5 and PE-Cy5.5 cannot be combined.

Optical detectors configuration detail_fortessa

Detector Array (laser)	PMT	LP Mirror	BP filter	Dye/detector
UV (355 nm) 20mW	A	505	530/30	Indo-1 (Blue)<br>*Hoechst Red 635LP 670LP<br>*BUV737 635LP 730/45
	B		450/50	HoeschstBlue<br>DAPI<br>Zombie UV<br>Live/Dead Blue<br>*Indo-1 Violet 405/20<br>*BUV396 379/28
	C			
Violet (405 nm) 50mW	A	750	780/60	BV786
	B	690	710/50	BV711
	C	635	670/30	BV650
	D	600	610/20	BV605
	E	505	525/50	BV510<br>BD Horizon V500<br>AmCyan
	F		450/50	Pacific Blue<br>BD Horizon V450

				BV421<br>Alexa 405<br>eFluor450<br>*CFP 480/30
Blue<br>(488 nm) 60mW	A	685	695/40	PerCP<br>PerCP-Cy5.5[2]
	B	505	530/30	FITC<br>GFP<br>YFP<br>Alexa 488<br>Venus<br>BB515
	C		488/10	SSC
Red<br>(640 nm) 40mW	A	750	780/60	APC-Cy7<br>APC-H7<br>APC-Alexa 750
	B	690	710/50	Alexa 700<br>APC-Cy5.5
	C		670/14	APC<br>Alexa 647
Yellow-Green<br>(561 nm) 50mW	A	750	780/60	PE-Cy7<br>PE-Alexa 750
	B	685	710/50	PE-Cy5.5[2]
	C	635	660/20	PE-Cy5<br>PE-Alexa 647<br>7AAD[1]
	D	600	610/20	PE-CF594<br>PE-Alexa 610<br>PI[1]<br>mCherry
	E		586/15	PE-Cy5.5[2]

3.4. Other Relevant Instrument Details
https://www.cincinnatichildrens.org/research/cores/flow-cytometry/analyzer
https://www.cincinnatichildrens.org/research/cores/flow-cytometry/software

4. Data Analysis Details

4.1. List-mode Data Files

FCS data files can be obtained by contacting Dr. Nives Zimmermann after this work has been published.

4.2. Compensation Description

Compensation has been performed at the time of acquisition, and checked computationally post-acquisition, according to the following spillover matrix (values in %):

Cantos/Immunophenotype

Show All	7-AAD-A	APC-A	APC-Cy7-A	BV421-A	BV605-A	FITC-A	PE-A	PE-Cy7-A
7-AAD-A	100	7	2.5	30	4.5	4	4	50
APC-A	0.7699	100	0	0.0513	0.1026	-0.154	0	0
APC-Cy7-A	0.546	28	100	0.2427	0.546	0.1214	0.182	4.6175
BV421-A	0.0023	0.0537	0	100	0.1331	0.1356	0.0163	0
BV605-A	8.1811	1.1307	0	20	100	-0.5384	2.8559	0.8614
FITC-A	1.5827	0.1356	0	0	0.1808	100	14.9168	0.1356
PE-A	11.7372	0.4766	0	1.0732	1.7658	2.0315	100	0.7626
PE-Cy7-A	0.3514	0.0836	3.7423	0.2175	0.2007	0.2594	1.4336	100

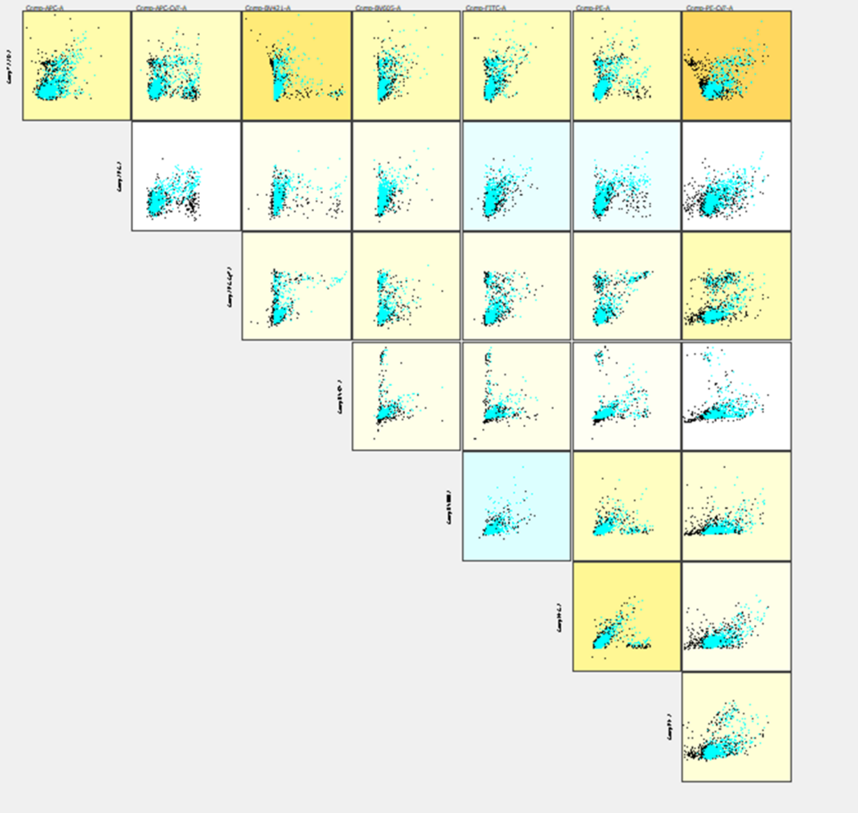

Cantos/activation status_ *In vivo*

Show All	7-AAD-A	APC-A	APC-Cy7-A	Alexa Fluor 700-A	BV421-A	BV605-A	PE-A	PE-Cy7-A
7-AAD-A	100	4.5694	1.4056	1.2338	-0.1527	5.4258	2.4409	4.2144
APC-A	-0.1899	100	10.5591	11.6451	0.4742	0.3476	1.1397	-0.2843
APC-Cy7-A	0.2368	6.7486	100	2.8301	0.1182	0.3014	0	0.3347
Alexa Fluor 700-A	12.8751	10.3036	104.1188	100	3.2965	6.2796	4.4967	1.0966
BV421-A	0.0666	0.0111	0	0.008	100	0.6984	0	0
BV605-A	16.7243	9.0988	3.3166	2.2695	9.2768	100	13.5234	1.5707
PE-A	11.4215	1.7663	0.1598	0.1537	0.4201	4.3988	100	0.2197
PE-Cy7-A	8.4423	5.4554	46.1416	1.3713	1.7592	4.5939	10.9145	100

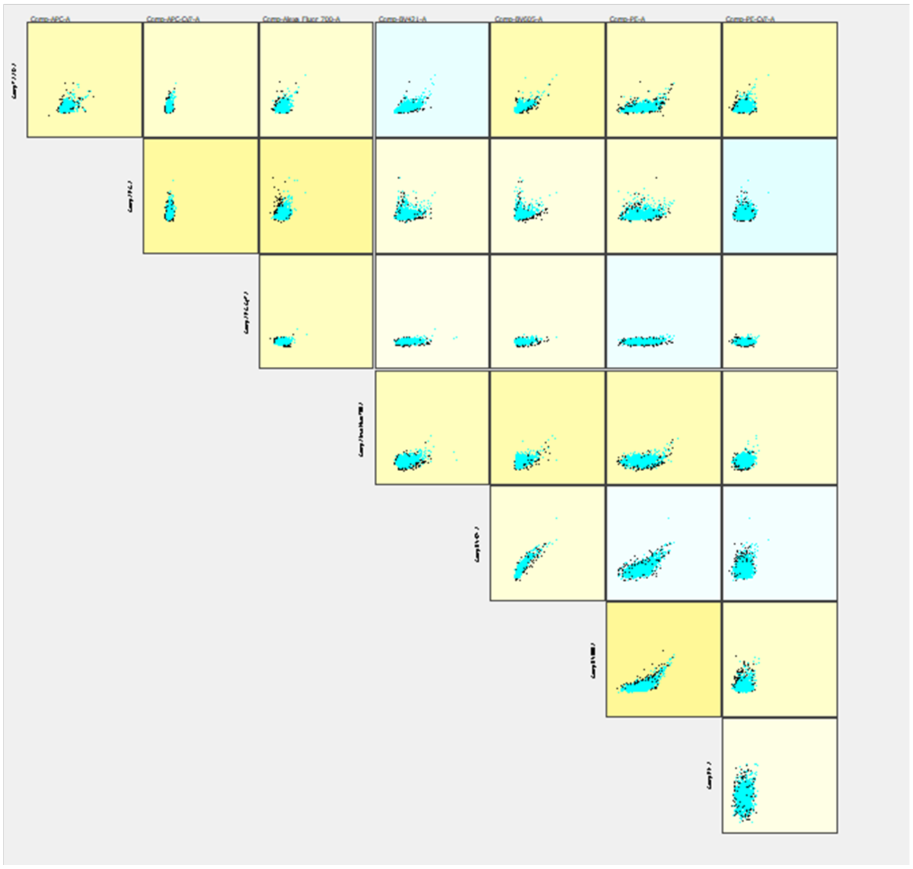

Fortessa/activation status_ *In vitro*

Show All	7-AAD-A	APC-A	Alexa Fluor 700-A	BV421-A	BV605-A	FITC-A	PE-A	PE-Cy7-A	APC-Cy7-A
7-AAD-A	100	30	10	0	0	20	12	0	0
APC-A	20	100	10	1.4383	0	-1	1.1495	0	0
Alexa Fluor 700-A	0	10	100	1.8658	15	0	1.5979	0	0
BV421-A	0	-0.2585	0	100	2	3	0	0	0
BV605-A	-50	7.8271	-12	40	100	-5	10	0	0
FITC-A	29	7	8	-16.5104	0	100	5	0	0
PE-A	12	0.0947	2	0.0112	0	0.8911	100	0	0.0112

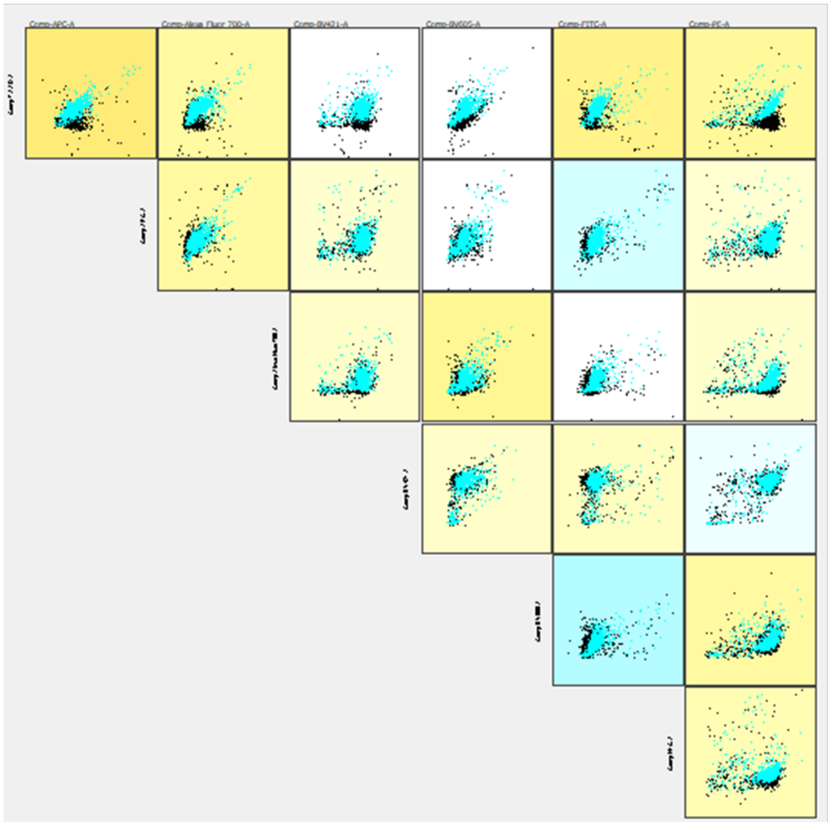

4.3. Data Transformation Details

4.3.1. Purpose of Data Transformation
Visualization and gating.
4.3.3. Other Relevant Data Transformation Details

- the default visualization setting in flowjo was used for gating:
- When digital data (fcs files) are brought into flowjo it automatically shows biexponentially transformed using logicle implementation.
- Scale: Biex
- Transforms:
  - Extra Negative decades:0
  - wideth basis:-100
  - Positive decades:4.42

4.4. Gating (Data Filtering) Details

The same gating strategy has been used for all data files (all challenged and unchallenged mice and/or stimulated and unstimulated BMDeos). For simplicity and

clarity, we provide details for only one sample gating; we include these as images within this document.

4.4.1. Gate Description

The gating strategy involves the following gates:

A.

- Total cells were gathered by FSC-SSC gating; this was called "cardiac cells"
- From the cardiac cell, "single cells" were selected by applying FSC-A/FSC-H gating (doublet exclusion)
- From "single cells", dead cells were removed, and the remaining cells are called "live cells"
- From "live cells", "CD45 positive" (hematopoietic) cells were selected
- From "CD45+", we identified B-cells (CD19+), T-cells (CD3+), Siglec-F+ and Ly6G+
- Activated eosinophils are a proportion of Siglec-F positive cells

B.

- Total cells were gathered by FSC-SSC gating this was called BMDeos
- From the BMDeos, single cells were selected by applying FSC-A/FSC-H gating and from singles cells
- Dead cells were removed by 7AAD, and the remaining cells are called viable or Live cells and from live cells
- Eosinophil (Eos) are determined by Siglec-F+/CCR3+
- Activated markers are gated out of eos

4.4.2. Gate Statistics

The following table shows an example of percentages of each of the subpopulations defined by described gates.

A

**ID# 3Wks T1**	**Total cardiac cells**	**Single cells**	**Live cells**	**CD45+**	**B cells**	**T cells**	**Siglec-F+**	**Ly6Ghi**
**% of FSC-SSC**	93.8							
**% of total cardiac cells**		94.4						
**% of single cells**			84.9					
**% of live cells**				37.3				
**% of CD45+**					2.68	2.77	37.3	26.5

B.

**ID# M1#1**	**Total cells**	**Single cells**	**Viable (7AAD-)**	**Eos (SigF+CCR3+)**
**% of FSC-SSC**	90.6			

**% of total cells**		99.2		
**% of single cells**			97.3	
**% of viable**				95.2

Gate Statistics applies to all used data files in a real experiment description. We only provide a single data file in order to keep this document as a clear and simple example.

4.4.3. Gate Boundaries

A.

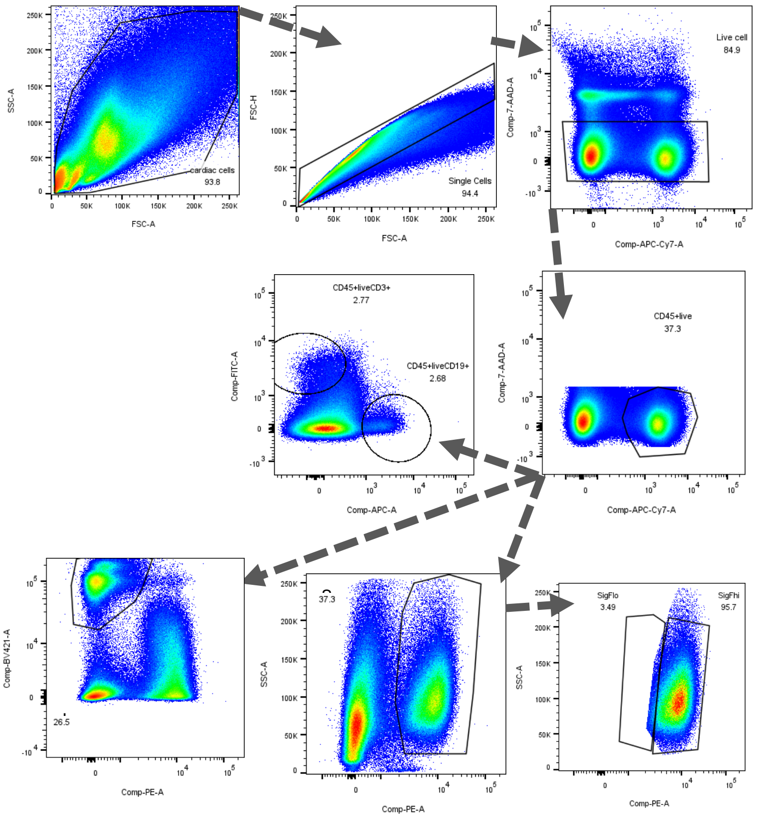

B.

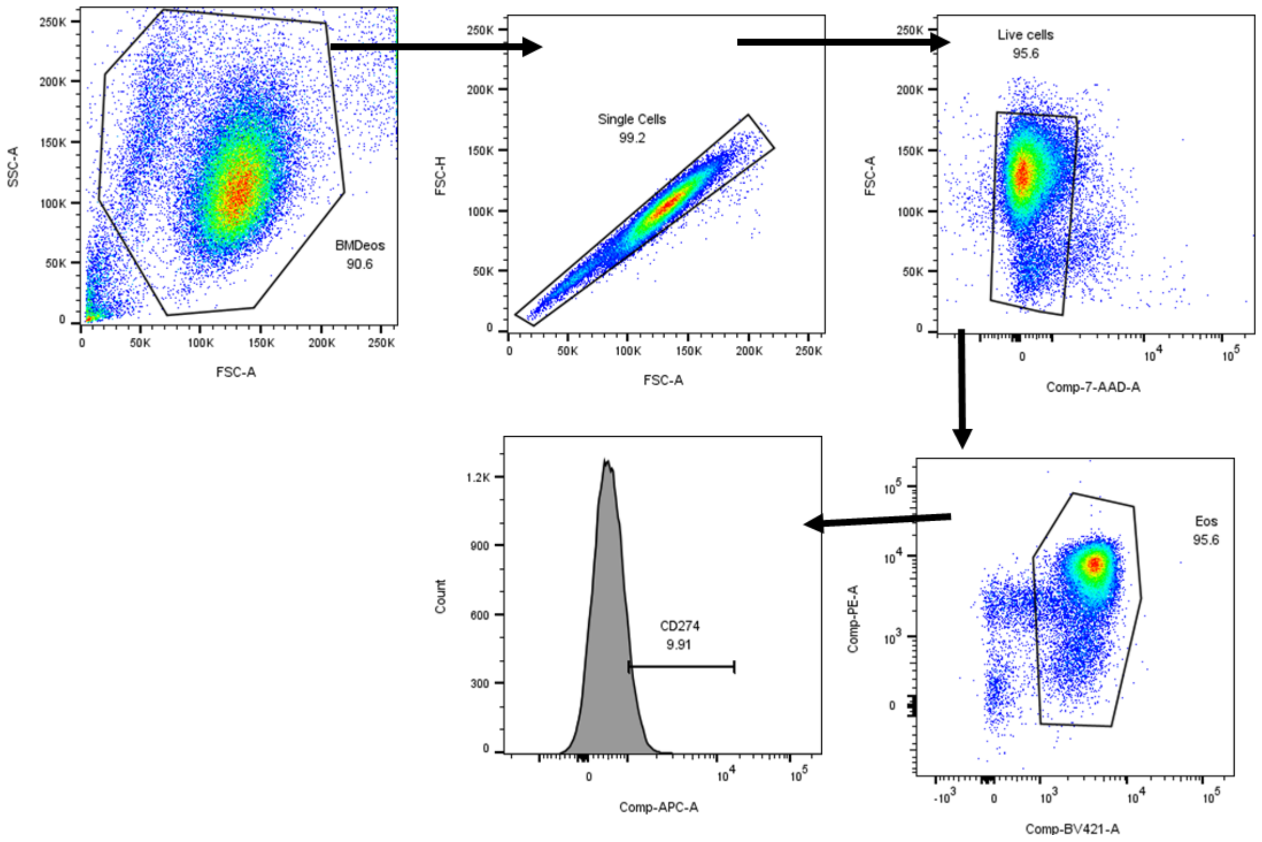

4.4.4. Other Relevant Gate Information

FlowJo workspace files could be obtained by contacting Dr. Nives Zimmermann after this work has been published.
